# Structural basis of 3′-end poly(A) RNA recognition by LARP1

**DOI:** 10.1093/nar/gkac696

**Published:** 2022-08-18

**Authors:** Guennadi Kozlov, Sandy Mattijssen, Jianning Jiang, Samuel Nyandwi, Tara Sprules, James R Iben, Steven L Coon, Sergei Gaidamakov, Anne M Noronha, Christopher J Wilds, Richard J Maraia, Kalle Gehring

**Affiliations:** Department of Biochemistry, McGill University, Montréal, Canada; Centre de recherche en biologie structurale, McGill University, Montréal, Canada; Intramural Research Program, Eunice Kennedy Shriver National Institute of Child Health and Human Development, National Institutes of Health, Bethesda, MD, United States; Department of Biochemistry, McGill University, Montréal, Canada; Centre de recherche en biologie structurale, McGill University, Montréal, Canada; Department of Biochemistry, McGill University, Montréal, Canada; Centre de recherche en biologie structurale, McGill University, Montréal, Canada; Centre de recherche en biologie structurale, McGill University, Montréal, Canada; Quebec/Eastern Canada NMR Centre, McGill University, Montréal, Canada; Intramural Research Program, Eunice Kennedy Shriver National Institute of Child Health and Human Development, National Institutes of Health, Bethesda, MD, United States; Intramural Research Program, Eunice Kennedy Shriver National Institute of Child Health and Human Development, National Institutes of Health, Bethesda, MD, United States; Intramural Research Program, Eunice Kennedy Shriver National Institute of Child Health and Human Development, National Institutes of Health, Bethesda, MD, United States; Department of Chemistry and Biochemistry, Concordia University, Montréal, Canada; Department of Chemistry and Biochemistry, Concordia University, Montréal, Canada; Intramural Research Program, Eunice Kennedy Shriver National Institute of Child Health and Human Development, National Institutes of Health, Bethesda, MD, United States; Department of Biochemistry, McGill University, Montréal, Canada; Centre de recherche en biologie structurale, McGill University, Montréal, Canada

## Abstract

La-related proteins (LARPs) comprise a family of RNA-binding proteins involved in a wide range of posttranscriptional regulatory activities. LARPs share a unique tandem of two RNA-binding domains, La motif (LaM) and RNA recognition motif (RRM), together referred to as a La-module, but vary in member-specific regions. Prior structural studies of La-modules reveal they are pliable platforms for RNA recognition in diverse contexts. Here, we characterize the La-module of LARP1, which plays an important role in regulating synthesis of ribosomal proteins in response to mTOR signaling and mRNA stabilization. LARP1 has been well characterized functionally but no structural information exists for its La-module. We show that unlike other LARPs, the La-module in LARP1 does not contain an RRM domain. The LaM alone is sufficient for binding poly(A) RNA with submicromolar affinity and specificity. Multiple high-resolution crystal structures of the LARP1 LaM domain in complex with poly(A) show that it is highly specific for the RNA 3′-end, and identify LaM residues Q333, Y336 and F348 as the most critical for binding. Use of a quantitative mRNA stabilization assay and poly(A) tail-sequencing demonstrate functional relevance of LARP1 RNA binding in cells and provide novel insight into its poly(A) 3′ protection activity.

## INTRODUCTION

The family of La-related proteins (LARPs) are RNA-binding proteins with multiple functions in the regulation of gene expression (1,2). Each subfamily of LARPs play specified and important functional roles in RNA metabolism. Phylogenetic analysis of LARPs, together with structural motif characteristics led to the classification of the LARPs into five distinct subfamilies: LARP1, LARP3 (genuine La protein), LARP4, LARP6, and LARP7. All LARPs share a highly conserved winged helix domain, termed the La motif (LaM), and in most cases this is associated with a member-specific downstream RNA recognition motif (RRM) (3). The tandem arrangement of LaM and RRM together is termed the La-module (4,5) with the domains connected by a variable interdomain linker region (6).

Despite their shared conserved features, different LARPs confer specific functions, mediated in part by family-specific domains and distinct RNA recognition by their La-modules (5). LARP1 regulates the stability and translation of mRNAs that encode components of the translation machinery, such as ribosomal proteins and translation factors (7–9). Known as TOP mRNAs, these contain a terminal oligopyrimidine (TOP) motif in the 5′ UTR, initiating with cytosine immediately after the 5′ m**^7^**Gppp cap (10). The TOP motif, comprised of 4–14 pyrimidines followed by a GC-rich region, allows for translational control of TOP mRNAs downstream of mTORC1 (8,11). LARP1 also binds to Raptor (7), a protein associated with mTORC1, and is directly phosphorylated by the mTOR kinase (10,12). Substantial data and multiple lines of evidence indicate LARP1 involvement with a large number of mRNAs in pathways related to cellular metabolism independent of mTOR and with important links to cancer (12–17). The C-terminal region of LARP1 contains a DM15 domain, which under conditions of mTORC1 inhibition, binds the mRNA 5′ m**^7^**G cap and TOP motif (11). This impedes the binding of eIF4E and obstructs formation of the translation initiation complex, thereby repressing TOP mRNA translation during unfavorable conditions (7,11,12,15). Whereas the DM15 region is essential for TOP mRNA regulation, an adjacent regulatory domain is necessary to prevent constitutive repression (10).

LARP1 and LARP4 have both been attributed with protection of poly(A) from deadenylation and associated mRNA stabilization. Both interact with poly(A)-binding protein (PABPC1) via a PABP-interacting motif 2 (PAM2) (reviewed in Mattijssen et al (2)). LARP4 differs from LARP1 in the position of the PAM2 relative to its La-module and RNA-binding sequences (18,19). Although the La-module of LARP4 plays a minor role in direct poly(A) binding relative to its N-terminal region (NTR), the RRM contributes more than the LaM (19). Studies indicate that disordered parts of the NTR drive RNA binding in a manner that suggests its conformational plasticity and of the La-module of LARP4 are important for RNA binding (6,19).

Structures of the LaM and RRM domains have been resolved for the human LARP3, LARP4, LARP6 and LARP7 (19–26). The LaMs and RRMs in these proteins form distinct but tethered structural domains that individually contribute in different ways in the different LARPs to the recognition of RNA targets (5,6,27). SAXS analysis of the La-modules of LARP3, LARP6, and LARP7 showed they adopt more compact structures upon RNA binding (6). The crystal structure of human LARP3 in complex with UUU_OH_ 3′ RNA revealed that LaM and RRM form a V-shaped clamp with the RNA ligand in the pocket of the LaM cleft interface (21,22,28). The 3′ penultimate uridylate makes direct contacts with both LaM and RRM domains, contributing to base specific recognition (28). The LaM and RRM domains of LARP6 and LARP7 also form a synergistic RNA binding scaffold, assisted by the participation of the flexible interdomain linker (5,21,23,29,30). The flexibility of La-modules as well as the presence of other RNA-binding domains gives rise to a significant degree of complexity in LARP RNA recognition and binding (1,22,23,27).

Despite the importance of LARP1 in regulation of ribosome biogenesis in response to mTORC1 signaling, its La-module remains poorly characterized with no structural information for the module either alone or in complex with RNA targets. Consistent with the association of LARP1 with PABP, LARP1 from human cell extracts immunoprecipitates with poly(A) RNA, but not with poly(U), poly(C), or poly(G) (31). High affinity of LARP1 La-module for poly(A) RNA was also observed in an electrophoretic mobility shift assay using purified LARP1 La-module (9,18). Assays in human kidney derived (HEK293) cells deleted of endogenous LARP1 (KO) showed that expression of LARP1 led to net lengthening of mRNA poly(A) tails (PATs) which was attributed to protection from deadenylation (18). Use of a classic β-globin reporter, containing the 38 nucleotide AU-rich element (ARE) of TNFα known to recruit the CNOT deadenylase, showed that PAT protection was linked to β-globin-ARE mRNA stabilization by LARP1 which was significantly decreased by point mutation to the PAM2 (see Figure 5 in (18)), whereas this mutation had no notable effect on the binding of A_15_ in electromobility shift assays (EMSA) using the purified LARP1 La-module (2).

In the present study, we use a variety of biophysical techniques to investigate the nature of the LARP1 La-module. Unexpectedly, we find that the module does not contain a RRM domain. NMR and ITC studies demonstrated the stand-alone LaM domain binds RNA with submicromolar affinity and preference for A-rich sequences. High-resolution crystal structures of LARP1 LaM in complex with poly(A) sequences of different length reveal the molecular basis for specificity for the RNA 3′-end and residues required for RNA binding. Using cellular assays of mRNA levels and poly(A) tail length, we demonstrate that high affinity binding by the LaM domain is critical for mRNA stabilization by LARP1.

## MATERIALS AND METHODS

### Expression and purification of proteins

Plasmids for bacterial expression of the LARP1 fragments 323–410, 323–417, 323–439, 323–509, 417–509 and 399–540 were obtained by mutagenesis of 310–540 fragment cloned into pET28a vector introducing deletions and stop codons at the appropriate positions using QuikChange Lightning Site-Directed Mutagenesis Kit (Thermo Fisher Scientific). LARP1 point mutants were obtained by site-directed mutagenesis using the QuikChange Lightning Site-Directed Mutagenesis Kit (Thermo Fisher Scientific). DNA sequencing was used to verify all sequence modifications. Proteins were expressed in *Escherichia coli* BL21(DE3) in rich (LB) medium as a fusion with N-terminal His-tag using induction with 1 mM IPTG for 4 h at 30 ºC. For NMR experiments, the recombinant protein was isotopically labeled by growth of *E. coli* BL21 in M9 minimal medium with ^15^N-ammonium sulfate as the sole source of nitrogen. Doubly labeled protein was prepared from cells grown in ^15^N-ammonium sulfate and ^13^C-glucose. Cells were harvested and broken in lysis buffer (50 mM HEPES pH 7.6, 0.5 M NaCl, 5% glycerol) containing 1 mM PMSF, 0.1 mg/ml lysozyme, 0.01 mg/ml DNase, 5 mM imidazole. The His-tagged proteins were purified by affinity chromatography on Ni^2+^-charged chelating Sepharose resin. The proteins were eluted with lysis buffer containing 500 mM imidazole. The resulting proteins contained an N-terminal MGSSHHHHHHS extension and were additionally purified and exchanged into the final buffer using size-exclusion chromatography using a HiLoad 16/600 Superdex 75 PG column (Cytiva) with HPLC buffer (10 mM MES pH 6.3, 100 mM NaCl, 1 mM TCEP).

### RNA oligonucleotides

A_25_, A_11_, A_6_ and C_2_UCU_4_C_2_GUG_2_CGC_2_UC were obtained from Sigma-Aldrich. A_2_UA_3_, A_2_CA_3_, ACA_4_, A_5_A(p), A_5_A(2′-OMe) were from Integrated DNA Technologies. Commercially obtained RNA samples were used without additional purification. A_3_, A_2_dA, A_4_, A_5_U, A_4_UA, A_3_UA_2_, A_5_C, A_4_CA, A_3_CA_2_ and additional samples of A_6_ and A_25_ were synthesized on 2 × 2 μmol scale using an ABI 3400 synthesizer with standard β-cyanoethylphosphoramidite chemistry on long chain alkylamine controlled pore glass (LCAA-CPG, 500 Å) with standard synthesis protocols. Oligonucleotides were purified either by preparatory denaturing PAGE or ion-exchange HPLC and desalted using C-18 SEP PAK cartridges as previously published (32).

### NMR spectroscopy

NMR samples were exchanged in 10 mM MES pH 6.3, 100 mM NaCl, 1 mM TCEP. Standard triple resonance (CBCACONH, HNCACB, HNCO) experiments on doubly (^15^N/^13^C) labeled protein were used to assign the backbone resonances. The assignments were deposited in Biological Magnetic Resonance Data Bank (BMRB) under accession number 51255. For NMR titrations, A_2_ and A_6_ RNAs were added to ^15^N-labeled LARP1 fragments to the final molar ratios of 1:2 or 1:1, respectively. Chemical shift perturbations were calculated as the weighted sum of proton and nitrogen shifts using the equation (ΔδH^2^ + (ΔδN/5)^2^)^1/2^. All NMR experiments were performed at 25 ºC using Bruker 600 MHz spectrometer. NMR spectra were processed using NMRPipe (33) and analyzed with SPARKY (34).

### Isothermal titration calorimetry

ITC experiments were performed on MicroCal iTC200 and VP-ITC titration calorimeters (Malvern Instruments Ltd). The syringe was typically loaded with 300 μM protein, while the sample cell contained 30 μM RNA (Supplementary Table S1). All experiments were carried out at 293 K with 19 injections of 2 μl with stirring at 310 rpm on iTC200 or 29 injections of 10 μl on VP-ITC. Results were analyzed using ORIGIN software (MicroCal) and fitted to a binding model with a single set of identical sites. The thermograms occasionally showed increased noise at later times, which we attribute to sample precipitation.

### Crystallization

Initial crystallization conditions were identified utilizing sitting drop vapor diffusion with the Classics II and Nucleix screens (QIAGEN). The best LARP1 LaM domain crystals were obtained by equilibrating a 0.6 μl drop of the protein (residues 323–410) at 20 mg/ml in HPLC buffer (10 mM MES pH 6.3, 100 mM NaCl, 1 mM TCEP), mixed with 0.6 μl of reservoir solution containing 0.2 M ammonium sulfate, 0.1 M Bis–Tris pH 6.5, 25% (w/v) PEG 3350. Crystals grew in 30–40 days at 20ºC. The best LARP1 LaM domain/RNA complex crystals were obtained by equilibrating a 0.6 μl drop of the LaM domain (residues 323–410) with oligonucleotide in a 1:1.1 molar ratio (10 mg/ml of protein) in buffer (10 mM MES pH 6.3, 100 mM NaCl, 1 mM TCEP), mixed with 0.6 μl of reservoir solution containing [0.056 M sodium phosphate, 1.344 M potassium phosphate] or [0.2 M sodium chloride, 0.1 M Bis–Tris pH 5.5, 25% (w/v) PEG3350] for A_3_, [0.056 M sodium phosphate, 1.344 M potassium phosphate] for A_4_, [0.1 M HEPES pH 7.5, 2 M ammonium sulfate] or [0.1 M Bis–Tris pH 5.5, 25% (w/v) PEG3350] for A_6_, [0.1 M BICINE pH 9.0, 1.6 M ammonium sulfate] for A_11_, [0.1 M HEPES pH 7.5, 25% (w/v) PEG 3350] for A_3_UA_2_. Crystals grew in 3–14 days at 20ºC. For data collection, crystals were cryo-protected by soaking in the reservoir solution supplemented with 30% (v/v) ethylene glycol in conditions using PEG3350 or with 25% glycerol otherwise.

### Structure solution and refinement

Diffraction data from single crystals of LARP1 LaM domain and its RNA complexes were collected at the Canadian Light Source (CLS), Cornell High-Energy Synchrotron Source (CHESS) and Advanced Photon Source (APS) (Supplementary Table S2). Data processing and scaling were performed with HKL2000 (35). The initial phases for the complex structure were determined by molecular replacement with Phaser (36), using the coordinates of the LARP3 LaM domain (PDB entry 1ZH5) (21). The initial phases were improved by Autobuilder in PHENIX package (37). The starting protein model was then completed and adjusted with the program Coot (38) and improved by several cycles of refinement, using the program phenix.refine (37) and model refitting. The resulting electron density maps revealed clear density for RNA oligonucleotide, which was manually built with the program Coot (38). The final protein model was then used for phasing of data for RNA-free LARP1 LaM domain. At the latest stage of refinement for both structures, we also applied the translation-libration-screw (TLS) option (39). The final models have all residues in the allowed regions of Ramachandran plot. The coordinates have been deposited with the Protein Data Bank (PDB). Refinement statistics are given in Supplementary Table S2.

### DNA constructs

The wild-type LARP1 cDNA corresponding to isoform-1 (1019 aa) version as in Mattijssen *et al.* (2) was subcloned into the HindIII and XbaI sites of the pFLAG-CMV2 vector (Sigma-Aldrich) and used as template for site-directed mutagenesis using the Q5 kit (NEB) to generate the Q333A mutant with primers: 5′- CATCAAGCGCGCTATTGAATACTACTTC and 5′-TAGTCTTTGAGCAGTTCC. The constructs were verified by bidirectional sequencing.

### Transfection

HEK293T LARP1 KO cells were a gift of Carson Thoreen (Yale University) and previously characterized for TOP mRNA and related translation factors (10). Transfections were carried out as described previously (18) with minor variations. The LARP1 KO 9 × 10^5^ cells per six-well plate were seeded one day prior to transfection with Lipofectamine 2000 (Invitrogen) according to the manufacturer's instructions. Because the LARP1 mutant accumulates to lower levels than wild-type LARP1 (not shown), more plasmid was added, and the difference was made up to 1 μg with empty pFLAG-CMV2 plasmid. For wild-type LARP1, 125 ng was transfected plus 875 ng empty plasmid; for LARP1-Q333A, 312.5 ng plus 687.5 ng empty plasmid. In addition, 100 ng of pcDNA3.1-β-globin−TNFα−ARE, 100 ng of pcDNA-TPGFP and 25 ng of pVA1 were cotransfected. 24 h post transfection, cells were split 1:4 into multiple wells, allowed to grow for another 24 h and then harvested for protein and RNA.

### Northern blotting

Cells were washed twice with 2 ml PBS per six-well, total RNA was isolated using Tripure (Roche) following manufacturer's instructions, except that the RNA pellet was washed 3 times with 1 ml 75% EtOH instead of once. Total RNA was then separated on a 1.8% agarose-formaldehyde gel and transferred to a GeneScreen-Plus membrane (PerkinElmer) overnight. After crosslinking with UV and vacuum-baking for 2 h at 80ºC, the membrane was prehybridized in hybridization solution (6× SSC, 2× Denhardt's, 0.5% SDS and 100 μg/ml yeast RNA) for one hour at hybridization incubation temperature (Ti). ^32^P-labeled oligo probes were added, and hybridization was overnight at Ti. Oligo probes used and their Ti can be found in Mattijssen et al (40). VA1 RNA was used as an internal control to normalize for mRNA recovery. Quantification statistics were calculated with GraphPad Prism software.

### Western blotting

Cells were washed twice with PBS and cell lysis was performed directly in RIPA buffer (Thermo Scientific) containing protease inhibitors (Roche). Proteins were size separated using SDS-PAGE and transferred to a nitrocellulose membrane. Primary antibodies used were anti-FLAG (Sigma, F1804), anti-actin (Thermo Scientific, PA1-16890) and anti-GFP (Santa-Cruz, sc-9996). Secondary antibodies from LI-COR Biosciences, which were conjugated to either IRDye 800CW or 680RD and western blot were scanned using the Odyssey CLx imaging system (LI-COR Biosciences).

### RNA-seq PAT length measurements

Single-molecule polyadenylated tail sequencing (SM-PAT-seq) was performed along with data handling and analysis as previously described (41,42). For these experiments, LARP1 KO cells were transfected as above, 2 × 6 wells per condition, but without pVA1 plasmid. 24 h post transfection, cells were split 1:4 into multiple wells, allowed to grow for another 24 h and then harvested for protein and RNA. RNA was purified using the Maxwell 16 LEV simplyRNA purification kit (Promega); cells were washed twice with 2 ml PBS per well and 100 μl homogenization buffer containing thioglycerol (Maxwell kit, Promega) was added per well to lyse the cells. Lysates from 6 wells per condition were combined in a tube and the Maxwell 16 LEV kit protocol was followed using 600 μl lysis buffer and three cartridges per sample (400 μl per cartridge). The total DNase-treated RNA was eluted in 40 μl H_2_O per cartridge. Protein was analyzed by western blot to confirm equal levels of wild-type and mutant FLAG-tagged LARP1.

## RESULTS

### LARP1 does not possess a classic tandem LaM-RRM module

Multiple members of the LARP super family from a variety of species were previously demonstrated to have tandem LaM and RRM domains with both engaged in RNA-binding activities (5). Although this has been widely assumed true, the initial characterization of the sequences of La-motif superfamily by examination of 134 sequences from a diversity of 29 eukaryotic species did reveal that LARP1 was an outlier in that 40% of LARP1 genes lacked a predicted RRM domain (3). In contrast, all the LARP3, 6 and 7 sequences, and 90% of LARP4 sequences were predicted to contain an RRM. Multiple LaM-containing proteins in yeast as well LARP1 homologs in *Arabidopsis thaliana, Caenorhabditis elegans, Danio rerio, Drosophila melanogaster, Escherichia coli, Homo sapiens, Mus musculus, Rattus norvegicus and Saccharomyces cerevisiae, Danio rerio, Drosophila melanogaster* and *Mus musculus, Schizosaccharomyces pombe elegans* and other metazoa were noted to lack an RRM ((43) and references therein).

**Figure 1.**
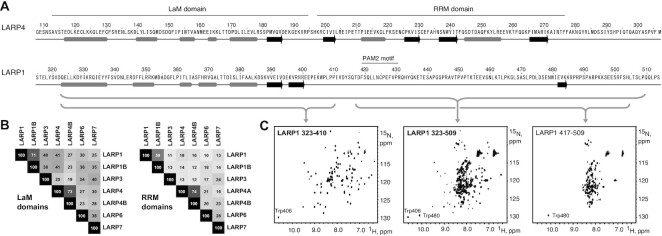
La-module of LARP1 does not contain an RRM domain. (**A**) Secondary structure predictions of the LaM and RRM domains of LARP4 and corresponding region of LARP1. Grey bars are alpha helices and black arrows are beta strands. LARP1 contains a PAM2 motif in the region corresponding to the LARP4 RRM but lacks predicted secondary structural elements. See also Supplementary Figure S1. (**B**) Sequence identity between the LaM and RRM regions of different human LARP proteins. (**C**) ^1^H–^15^N NMR correlation spectra of ^15^N-labeled LARP1 fragments (numbered according to the 1019-residue long isoform). The spectrum of residues 323–509 (middle spectrum) shows a mix of dispersed signals typical of a folded domain and a central cluster typical of an unfolded protein. Spectra of the separate N- and C-terminal halves confirms that residues 323–410 adopt a folded structure while residues 417–509 are unstructured. See also Supplemetary Figures S2 and S3.

Secondary structure prediction of human LARP1 with JPred4 (44) shows the absence of secondary structure elements in the region corresponding to the LARP4 RRM (Figure 1A and Supplementary Figure S1). AlphaFold similarly failed to predict a folded domain for this region of human LARP1 and LARP1B, as well as for these proteins from other species examined including chicken, frog, zebra fish, mouse and rat, whereas all examples of LARPs 3, 4, 6 and 7 were predicted to have an RRM (45). Sequence comparisons of the LaM and RRM regions of the human LARPs reveals greater similarity of the LaMs whereas the RRMs are more divergent (Figure 1B), in agreement with the analyses in other species (3).

To address this experimentally, we analyzed LARP1 fragments containing both domains by NMR spectroscopy. The constructs were expressed in bacteria and ^15^N-labeled proteins purified for NMR spectroscopy. The ^1^H–^15^N correlation spectrum of residues 323–509 encompassing the LaM-RRM (numbered according to the 1019-residue long isoform) showed a mixture of weaker, dispersed signals and a central band of intense signals (Figure 1C, middle panel). This is characteristic of the presence of a mix of ordered and disordered residues with the dispersed signals arising from a folded domain and the strong signals coming from unfolded residues. This fragment contains two tryptophans: Trp406 in the predicted LaM and Trp480 from the putative RRM. To confirm that the well-dispersed signals corresponded to the LaM domain, we produced separate constructs of the LaM and RRM regions. ^15^N-labeled LARP1 (323–410) yielded a well-dispersed spectrum that closely matched the weaker signals in the (323–509) NMR spectrum including the Trp406 indole proton (Figure 1C). In contrast, ^15^N-labeled LARP1 (417–509) representing the putative RRM alone, produced a spectrum with poorly dispersed signals that matched the unfolded set of signals in the 323–509 fragment (Figure 1C). To rule out the possibility that the predicted RRM had been prematurely truncated, removing residues required for proper folding, we examined a larger fragment terminating at residue 540. The LARP1 (399–540) spectrum retained the characteristics of an unfolded protein and showed no changes in the presence of A_25_ RNA (Supplementary Figure S2). Examination of additional fragments identified residues 323 and 410 as the boundaries of the LARP1 LaM domain (Supplementary Figure S3).

### The stand-alone LaM domain is sufficient for poly(A) binding

**Figure 2.**
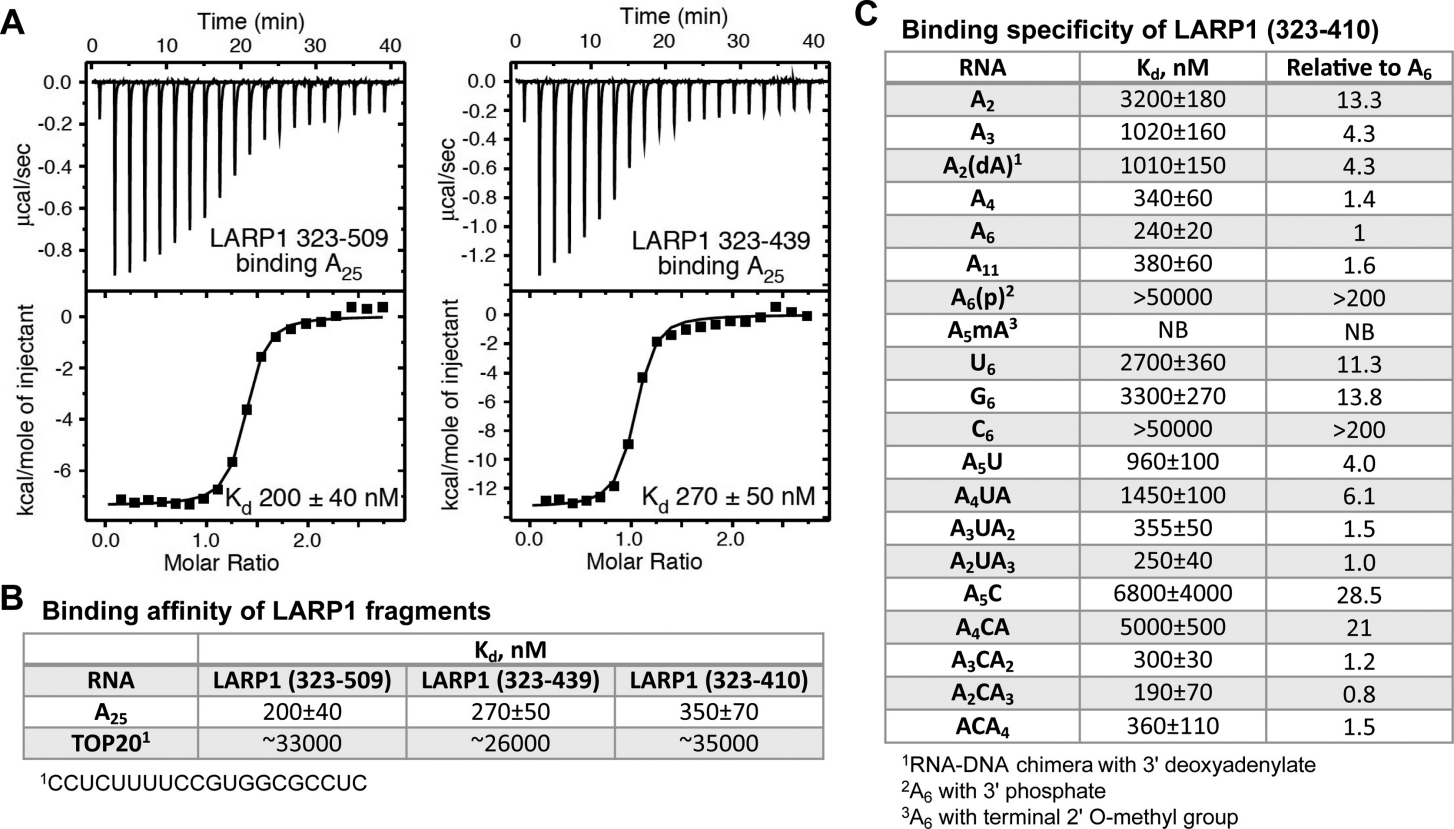
Isothermal titration calorimetry of RNA binding to LARP1 LaM domain. (**A**) ITC thermograms of LARP1 constructs binding to A_25_. The RRM region is dispensable for full affinity binding. (**B**) Affinities of LARP1 fragments binding poly(A) and a TOP RNA. The LaM domain binds A_25_ with high nanomolar affinity but only micromolar affinity for a 20-mer TOP RNA. (**C**) Affinities of different ligands confirms specificity of the LaM domain for the 3′ end of poly(A) RNA. See Supplementary Table S1 and Supplementary Figure S4 for a complete listing of the ITC experiments.

We used isothermal titration calorimetry (ITC) to measure the affinity of the LARP1 La-module for RNA. ITC thermograms with LARP1 (323–509) and A_25_ allowed us to determine *K*_d_ of ∼0.2 μM (Figure 2A) with a stoichiometry close to 1:1 (Supplementary Table S1 and Figure S4). Similar affinities were observed for the smaller fragment, LARP1 (323–439) that encompasses the LaM domain and the PAM2 motif (Figure 2A, B), and LARP1 (residues 323–410) comprising the LaM domain without PAM2 (Figure 2B). These data confirm that the LARP1 LaM domain is sufficient for poly(A) binding with submicromolar affinity. We next measured the affinity of LARP1 fragments for binding a previously characterized TOP RNA sequence. The intact LaM-RRM 323-509 fragment had only weak affinity for the TOP RNA (*K*_d_ of 33 μM) with little difference compared to LaM 323–439 or 323–410 (Figure 2B). This contrasts with LARP3 in which previous studies identified a crucial role for both domains of the La-module for poly(U) binding (21,22).

### LARP1 LaM recognizes the 3′nucleotides of poly(A)

We carried out additional ITC experiments to determine ligand binding specificity of the LaM. Comparison of shorter oligonucleotides showed that three or four nucleotides were sufficient to capture most of the binding affinity (Figure 2C). Notably, A_11_, A_6_ and A_4_ bound with roughly the same affinity as A_25_. The small variations in affinity and binding stoichiometry are due to uncertainties in the ligand concentration and a slight tendency of the LARP1 (323–410) fragment to precipitate during ITC experiments.

The 1:1 stoichiometry observed with A_25_ suggest an absence of LaM binding to the middle nucleotides of the poly(A) RNA. Specificity for the 3′-end was determined by experiments with RNAs modified at the 3′-ribose. Addition of a 3′ phosphate on A_6_ led to a 200-fold loss of affinity (Figure 2C). Similarly, 2′-*O* methylation of the 3′ nucleotide of A_6_ completely blocked binding. The effect of the phosphate or methyl group appeared to be steric as replacement of the 3′ nucleotide in A_3_ with a DNA nucleotide (dA) had no effect on *K*_d_ (Figure 2C). Similar results were observed with LARP3 (21,22,28), where the LaM domain mediates binding of the 3′-end nucleotides of poly(U).

### Specificity for poly(A) RNA binding by the LARP1 LaM domain

To examine base specificity of RNA binding, we used ITC to measure affinities of the LaM domain for A_6,_ U_6_, G_6_ and C_6_ (Figure 2C). LARP1 LaM had the highest affinity for A_6_ with a 10-fold lower affinity for U_6_ and G_6_ RNA. Cytosine bases were very poorly tolerated as C_6_ RNA showed more than a 200-fold lower affinity relative to A_6_ (Figure 2C). We also performed nucleotide scanning binding experiments to determine positional specificity of the LaM domain. These showed that uracil substitutions were generally tolerated with a 3- to 4-fold decrease in binding affinity at positions –1 and –2 and no effect at –3 (Figure 2C). In contrast, C-scanning showed larger, 10-fold losses in affinity at positions –1 or –2, which together account for most of the effect observed with C_6_. Cytosine substitutions at positions –3, –4 and –5 had little effect on binding (Figure 2C). Taken together, the U- and C-scanning experiments confirm that the LaM domain specifically recognizes the 3′-end of poly(A) RNA.

### NMR studies of poly(A) RNA binding to LARP1 LaM in solution

**Figure 3.**
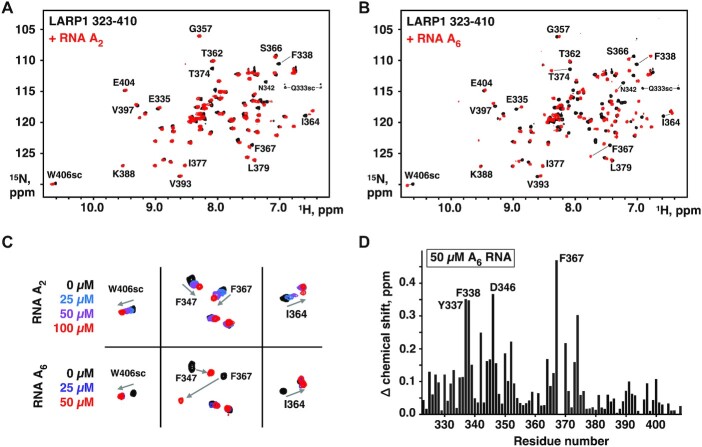
NMR of RNA binding. (**A**) Spectra of 50 μM ^15^N-labeled LARP1 (323-410) alone (*black*) and in the presence of 100 μM A_2_ (*red*). Shifts of selected signals are labeled. Side chains resonances are labeled *sc*. Signal assignments are shown in Supplementary Figure S5. (**B**) Spectra of 50 μM ^15^N-labeled LARP1 (323–410) alone (*black*) and in the presence of 50 μM A_6_ (*red*). (**C**) Comparison of peak shifts showing fast-exchange in the A_2_ spectra and slow-exchange in the A_6_ spectra. The slower dynamics of A_6_ binding is consistent with higher affinity of A_6_. (**D**) Plot of LaM amide proton and nitrogen chemical shifts changes upon A_6_ binding. Supplementary Figure S6A shows the shifts mapped onto the 3D structure.

We next turned to NMR to characterize the RNA-binding site on the LaM domain. ^13^C,^15^N-labeled protein was prepared, and the NMR signals of LARP1 (323–410) assigned using standard triple resonance techniques (Supplementary Figure S5). We carried out two titrations acquiring NMR spectra of the LaM domain in the presence of increasing concentrations of the dinucleotide A_2_ and the hexamer A_6_ (Figure 3). Both RNAs caused peak shifts in the ^1^H–^15^N correlation spectra confirming binding. The shifts were generally larger for A_6_, consistent with higher affinity binding; the most significant difference was in the increased dynamics of binding observed with A_2_. Intermediate titration point with A_2_ showed fast-exchange between the free and bound states with peaks sliding between the initial and final positions. Several peaks, such as Phe338, showed exchange broadening and disappeared during the titration. In contrast, the signals corresponding to the free and bound states in the A_6_ titration with were in slow-exchange as is typical for sub-micromolar binding affinity (Figure 3C). The higher affinity of A_6_ was also apparent in the amount of RNA required to reach saturation. A_2_ required over-titration to a 1:2 ratio of protein to RNA, while 1:1 was sufficient for A_6_.

We used triple resonance and ZZ-exchange experiments to assign the signals for the LaM-A_6_ complex. The amide resonances of Tyr337, Phe338, Asp346 and Phe367 showed the largest shifts upon RNA binding (Figure 3D). Plotting the chemical shift changes (as the weighted average of ^1^H and ^15^N shifts) shows most of affected residues are in the N-terminal half of the domain.

### Structure of the LARP1 LaM domain

**Figure 4.**
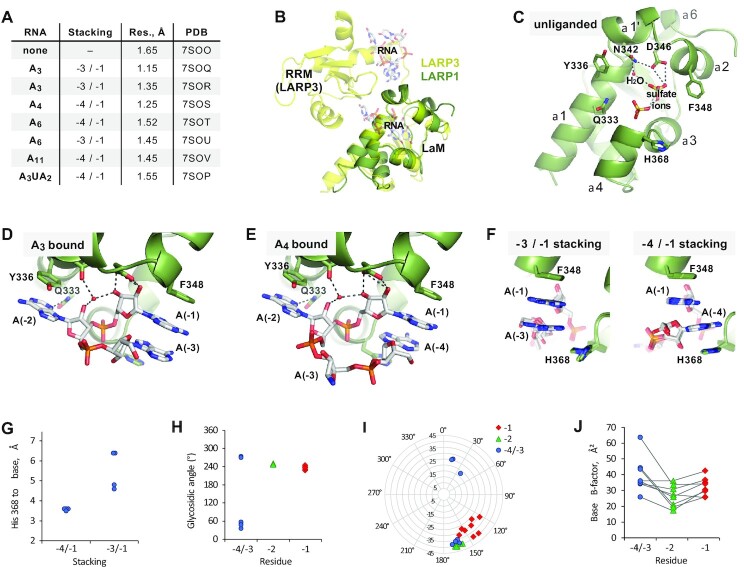
Structures of LARP1 LaM domain in complex with poly(A) RNA oligonucleotides. (**A**) Table of eight crystal structures reveals two different base stacking configurations. Supplementary Figure S7 shows the individual structures. (**B**) Overlay of LARP1 LaM (*green*) with La module of LARP3 (*yellow*; PDB 2VOD). RNA bound to the LARP3 LaM and RRM domains is shown semi-transparent. (**C**) Structure of unliganded LaM. A water molecule and two sulfate ions occupy the RNA binding site and are part of a conserved hydrogen bonding network (*dashed lines*) formed by Asn342 and Asp346. (**D**) Structure of LaM with A_3_ RNA bound (nucleotides numbered from the 3′-end). Asn342 and Asp346 are responsible for binding of the RNA 3′-end. Adenine bases (–1) and (–3) stack together adjacent to Phe348, while A(–2) stacks against Tyr336. (**E**) Structure of LaM with A_4_ bound reveals an alternative stacking configuration where bases A(–1) and A(–4) stack together and A(–3) is disordered. (**F**) Comparison of the two different base stacking configurations in the seven structures. His368 contributes to the stability of the –4/–1 stacking configuration (four structures) by stacking against the A(–4) adenine ring. (**G**) Inter-ring distances between His368 and the A(–4) base. (**H**) Glycosidic angles of the bound RNAs. Six RNAs contained *syn* angles (χ around 60º) for nucleotides A(–4) or A(–3). The angles of the A(–2) nucleotides varied by less than 5º. (**I**) Ribose pucker and pseudorotation angles. The riboses of A(–1) and A(–2) were largely C2′-*endo* but three riboses of A(–3)/A(–4) were C3′-*endo*. (**J**) *B*-factors of the adenine bases show the A(–1) and A(–2) nucleotides are the most highly ordered.

We employed X-ray crystallography to investigate the molecular mechanisms responsible for the binding specificity of LARP1 (Figure 4, Supplementary Table S2). Crystallization trials with LARP1 (323–417) yielded fast-growing crystals in multiple conditions but the best crystals were obtained with LARP1 (323–410). These diffracted to better than 2 Å and allowed the structure of the unliganded protein to be solved using molecular replacement with the LaM domain of LARP3 (21). The DALI server (46) identified LARP7 (PDB 4WKR; 1.1 Å RMSD) and LARP3 (PDB 1S29; 1.2 Å RMSD) as the closest structural homologs (29,47).

Unsurprisingly, the structure is highly similar to previous LaM module structures but without the RRM domain (Figure 4B). One consequence is that the extended linker between the LaM and RRM domains in the previous structures forms a short helix (α6) in LARP1 (Figure 4C). The LARP1 structure contains bound sulphate ions, which frequently are found in phosphate-binding sites. The two most ordered sulfates are stabilized by a network of ionic interactions from residues Asn342 and Asp346 and a well-ordered water molecule (Figure 4C). The residues around the sulfates are well-conserved across the family of LARP proteins and include an abundance of positively charged residues (Supplementary Figure S6). Overlaying the LARP1 and LARP3 structures confirms that the sulfates superimpose with the RNA-binding site in LARP3 (Figure 4B). Mapping of the NMR chemical shift changes onto the crystal structure shows the residues with the largest NMR peak shifts upon RNA binding (e.g. Tyr337, Asn342, Asp346, Phe367) are located at the same site (Supplementary Figure S6).

### Structural determinants of poly(A) RNA binding

Crystallization trials with RNAs between three and eleven nucleotides in length produced seven complex structures, including non-isomorphic structures with the same ligand (Figure 4A, Supplementary Figure S7, Supplementary Movies S1 and S2). The crystals show density only for the 3′-terminal nucleotides and can be classified into two groups based on the stacking of the first and last base. The LaM appears to act as a rigid scaffold and the differences between the structures largely arise from differences in the conformation of the bound RNA. To facilitate comparison of the complexes, the nucleotides are numbered from the 3′-end: A(–1) to A(–4). The highest resolution structure (1.15 Å) was observed with the complex containing A_3_ (Figure 4D). The O3′ and O2′ of the ribose of the 3′ terminal nucleotide, A(–1), makes hydrogen bonds with Asp346, and the O2′ also makes hydrogen bond contact with a water molecule, collectively replacing one of the sulfate ions in the apo structure. This water molecule was observed in all the structures including the unliganded structure. The A(–1) adenine ring packs against the side chain of Phe348, and its phosphate sits along the axis of the α4 helix dipole with hydrogen bonds to the side chain of Tyr337 and the backbone amide of Arg369 (not shown). Although the ITC results show a preference for an adenine base, there are no hydrogen bonds between LaM domain residues and the 3′ base. Instead, the specificity appears to result from base stacking and hydrogen bonds to bound waters.

The A(–2) nucleotide ribose O2′ makes a hydrogen bond with the ordered water while the adenine base is flipped out and away from the 3′-nucleotide. The A(–2) base stacks against the side chain of Tyr336 and the adenine N3 forms a hydrogen bond with the side chain of Gln333, which may contribute to the base selectivity. The A(–3) nucleotide is flipped back where it stacks against the A(–1) base. The phosphate between the –2 and –3 nucleotides is stabilized by Arg345 (Supplementary Figure S6). This stacking arrangement (–3/–1) was observed in three of the seven liganded structures.

In the crystal with A_4_ bound (Figure 4E), the positions and contacts of 3′ terminal nucleotides A(–1) and A(–2) are almost identical to those in the A_3_ crystal; however, the A(–3) nucleotide is partially disordered and does not make any contacts with the LaM. Instead, the nucleotide A(–4) stacks on A(–1). This stacking arrangement (–4/–1) was observed in four of the seven ligand structures (Figure 4F). The –4/–1 stacking benefits from an additional contribution from the side chain of His368 which stacks on the base of A(–4). The two classes of structures could also be distinguished by the shorter distance between the His368 side chain and the adenylate base in the –4/–1 configuration (Figure 4G). The –3/–1 stacking was not unique to the short length of the A_3_ ligand as this configuration was also observed in one of the A_6_ structures (Figure 4A). The altered stacking arrangements is reminiscent of poly(U) sequences interacting with LARP3, in which U(–1) stacks on U(–3) or alternatively U(–1) stacks on U(–4) (22).

Analysis of the nucleic acid backbone angles confirmed the rigidity of 3′-end of poly(A) RNA, which results from tight binding to the LaM domain (Figure 4H–J). The glycosidic angles and sugar puckers for A(–1) and A(–2) were tightly constrained with A(–2) generally showing the smallest spread in structural parameters and lowest *B*-factors. In contrast, a large range of angles, including *syn* glycosidic angles and C3′-*endo* sugar puckers, were observed for A(–3) and A(–4) nucleotides (Supplementary Table S3). The structural heterogeneity suggests fewer structural constraints and weaker binding of these nucleotides.

### Mutagenesis confirms the interactions observed in crystal structures

**Figure 5.**
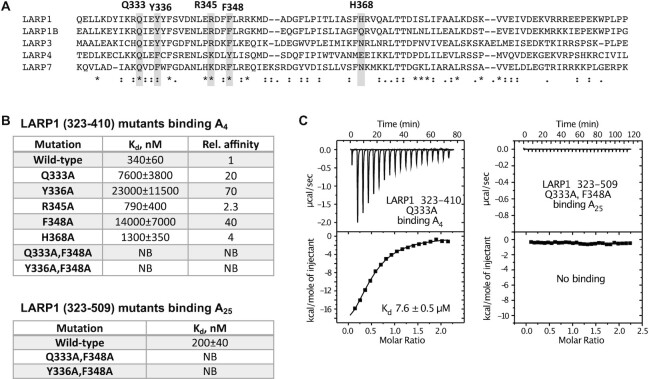
Mutagenesis of key LaM residues for poly(A) binding. (**A**) Sequence alignment of LaM domains with the amino acid residues selected for mutagenesis. (**B**) ITC results reveal reduced affinity of the Q333A, Y336A and F348A LaM mutants, and complete loss of binding for Q333A/F348A and Y336A/F348A double mutants. The mutations also prevented binding of A_25_ RNA in the context of the larger LARP1 fragment (323-509). (**C**) ITC thermograms of Q333A and Q333A/F348A mutants.

We produced point mutants of the LaM (323–410) and tested their affinity for RNA by ITC (Figure 5). We verified that the mutants were correctly folded by isotopically labeling them and recording their ^1^H–^15^N correlation spectra. All the mutants yielded NMR spectra similar to the wild-type protein consistent with small perturbations restricted to the environs of the mutations (Supplementary Figure S8). Single point mutants in the binding site decreased the binding affinity of A_4_ by one to two orders of magnitude with loss of aromatic residues having the largest effects (Figure 5). Loss of Gln333, which makes a hydrogen bond to the A(–2) adenine base, had a surprisingly large effect and decreased binding affinity 20-fold. Conversely, Arg345 plays a relatively minor role in binding as its mutation to alanine only decreased the affinity by 2-fold.

As the single-point mutants Q333A, Y336A and F348A still displayed weak RNA binding, we generated and tested two double mutants, Q333A/F348A and Y336A/F348A. Both double mutations completely abolished binding to A_4_ (Figure 5B). We also tested the double mutants in the context of the longer LARP1 fragment that contains both the LaM and the supposed RRM. The mutations again prevented RNA binding, ruling out a role of the RRM region in poly(A) RNA binding (Figure 5C).

### LARP1 *in vivo* assay for poly(A) protection and mRNA stabilization

We previously developed an *in vivo* assay in LARP1 knock-out (KO) cells to monitor the ability of LARP1 to stabilize a widely used type reporter β-globin mRNA containing a 38 nucleotide AU-rich element (ARE) of instability in its 3′ UTR that was taken from the TNFα mRNA (18). The TNFα ARE is a high affinity binding motif for the tristetraprolin protein (TTP or ZFP36) that recruits the CNOT deadenylase to mRNAs and leads to their instability (48,49). Insertion of this ARE into the β-globin reporter (β-glo-ARE) mRNA decreases its half-life (*t*_1/2_) from several hours to 75–90 min (50). Differences in the steady state levels of the β-glo-ARE mRNA detected by northern blot reflect the poly(A) protection and mRNA stabilization activities of different LARP4 or LARP1 mutant proteins examined by transient transfection experiments (18,40). The assay includes a co-transfected GFP reporter which provides a way to monitor poly(A) tail (PAT) protection of a stable mRNA. The GFP mRNA half-life is 6–8 h and it is highly expressed. Analysis of the two mRNAs provides a large window into the effects of LARP1 on mRNA stability (2,18).

**Figure 6.**
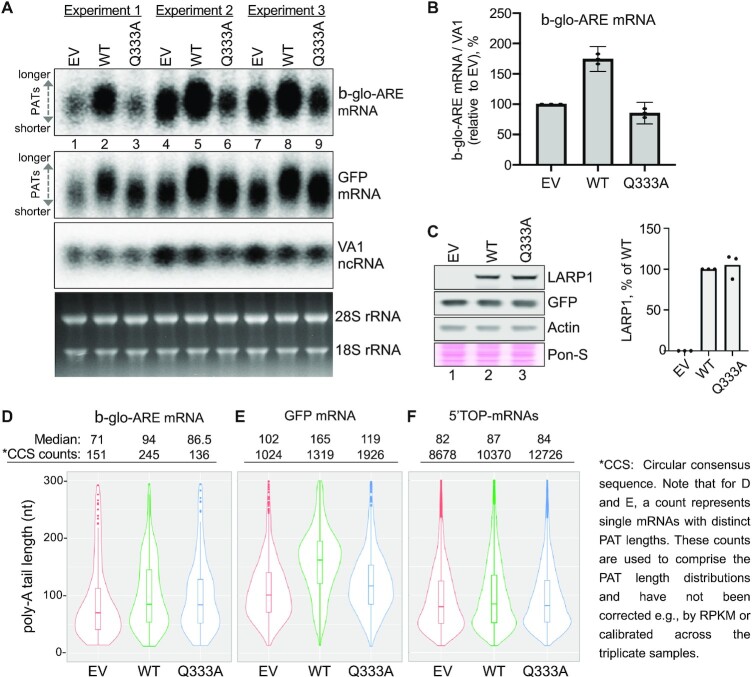
RNA-binding by the LARP1 LaM is required for PAT protection and mRNA stabilization. (**A**) Northern blot analysis of total RNA isolated from HEK293T cells 48 hours post transfection with the constructs indicated above the lanes: EV = empty vector, WT = wild type LARP1, Q333A = RNA-binding defective mutant. Samples from three independent experiments were performed and analyzed on one blot. The probes used are indicated to the right of the panels. Bottom panel shows an image of the EtBr-stained gel before transfer. (**B**) Quantitation of the β-globin-ARE mRNA signals from the northern blot in (A) normalized by the VA1 signals for replicate biological experiments; *N* = 3, error bars represent the 95% interval. (**C**) Western blot (left) and quantification of protein levels (right) of the three experiments in panel A. The blot was probed with antibodies against LARP1, GFP and actin. Ponceau S (Pon-S) was used to stain total protein. LARP1 levels were normalized by actin. (**D–F**) Results of SM-PAT-seq analysis combined from three independent transfection experiments. The PAT lengths obtained by SM-PAT-seq are represented by violin plots in which the rectangles show the 95% confidence interval. The median PAT lengths above the plots are the circular consensus sequence (CCS) read counts (each CCS count represents an mRNA molecule with a specific PAT length). (D) PAT data for β-glo-ARE mRNA. (E) PAT data for GFP mRNA. (F) PAT data for 97 endogenous 5′ TOP mRNAs (51).

Full length LARP1 or La-module fragments of LARP1 were shown to stabilize β-glo-ARE mRNA using the quantitative blot assay, and this was diminished by point mutations to the LARP1 PAM2 motif (18). For the present study, we again used the β-glo-ARE reporter to test the importance of RNA binding by the LARP1 LaM in the quantitative mRNA stabilization assay (Figure 6). We examined effects of the full length LARP1-Q333A mutant. This single point mutation of a conserved glutamine in the LaM led to a 20-fold decrease in RNA binding with no effect on folding of the domain (Figure 5; Supplementary Figure S8). The HEK293T LARP1-KO cells were transfected with empty vector (EV), Flag-tagged LARP1 (wild-type, WT) or Flag-tagged LARP1-Q333A. The cells were cotransfected with aliquots of a mixture of expression plasmids for β-glo-ARE, GFP, and the VA1 small noncoding RNA transcribed by RNA polymerase III, the latter as a transfection control. Forty-eight hours after transfection the cells were harvested, and RNA examined to assess mRNA levels and PAT length (Figure 6A, B). In each of the three replicate experiments, cells transfected with LARP1 WT accumulated higher levels of mRNAs for β-glo-ARE relative to EV and LARP1-Q333A. Further, there was an upward mobility shift indicative of increased PAT length in the LARP1-WT cells, consistent with 3′ end protection. To confirm that the effects were not due to differences in LARP1 expression, we immunoblotted the cell extracts and observed equal LARP1 levels in the WT and Q333A expressing cells (Figure 6C). The same northern blot probed for GFP mRNA provided separate evidence of PAT protection by LARP1-WT that is lacking for LARP1-Q333A (Figure 6A). These gels yield better resolution of the differences in PAT length since, as shown below, the PATs of GFP mRNAs are longer than for β-glo-ARE mRNA. In contrast, the effect of the Q333A mutation on GFP mRNA levels was smaller than observed with the β-glo-ARE mRNA.

### Poly(A) tail protection by LARP1

We used single-molecule sequencing to measure PAT lengths (41,42). Total RNA was prepared from LARP1 KO cells transfected as for northern blot analysis. Splinted-primer adapters were ligated to poly(A) 3′ ends and used to synthesize first strand cDNA followed by random hexamers to synthesize complementary strands. The amplified library of 1–2 kb cDNAs was sequenced using a PacBio Sequel system and analyzed to obtain the PAT lengths of different mRNAs.

Comparison of PAT length distributions obtained by PAT-seq for β-glo-ARE and GFP confirmed the PAT length increase observed on northern blots in LARP1-WT cells relative to Q333A and EV (Figure 6D, E). A major difference between the β-glo-ARE and GFP mRNAs is the steady state PAT lengths in the EV cells as well as in the LARP1-WT and -Q333A cells. The violin plots reveal a higher fraction of β-glo-ARE mRNAs with shorter PATs as compared to GFP (compare EV in Figure 6D to EV in 6E), consistent with recruitment of CNOT by the ARE. The β-glo-ARE mRNA median PAT length was shifted from 71 nt in EV cells to 94 nt in LARP1-WT cells as the fraction of mRNAs with lengths extended to near 200 nt increased (Fig 6D). At 86.5 nt median length, the β-glo-ARE PATs in LARP1-Q333A cells were intermediate between EV and LARP1-WT (Figure 6D). Strikingly however, this increased median PAT lengthening by LARP1-Q333A did not lead to a corresponding or any stabilization of the β-glo-ARE mRNA (Figure 6B).

We had previously provided evidence for LARP1 PAT protection of rpL35 mRNA encoding ribosomal protein L35, using the gel mobility assay (18). For the present analysis, examination of the pool of 5′ TOP mRNAs (51) revealed a relatively small increase in their median PAT lengths (Figure 6F). The Q333A mutant had an intermediate effect on the 5′ TOP mRNA PAT length but the difference was too small to claim statistical significance. In conclusion, these experiments show that RNA binding by the LaM domain is required for LARP1′s poly(A) protection and mRNA stabilization activity in cells.

## DISCUSSION

While La-modules in other LARP family members have been characterized structurally and functionally, the LARP1 La-module has remained understudied. Our study closes this gap and reports the detailed molecular basis of RNA binding by the LaM domain present in the LARP1 La-module. We provide compelling evidence that human LARP1 does not possess an RRM domain following its LaM domain and suggest this is most likely the case for LARP1B based on its sequence similarity to LARP1. Although a folded domain could not be identified in this region, work from ourselves and other suggests that the region makes functional contributions to RNA recognition and stabilization (9,18).

We used ITC and NMR spectroscopy to demonstrate that the LARP1 LaM can function as a stand-alone RNA-binding domain with submicromolar affinity for poly(A) RNA. ITC experiments showed that the LaM specifically recognizes the 3′-end of RNA with a clear preference for A-rich sequences (Figure 2). Poly(C) showed no binding nor did RNA oligomers without a free 3′ hydroxyl. U- and C-scanning experiments showed that the sequence specificity is restricted to the two nucleotides at the 3′-end. In ITC experiments, dinucleotide A_2_ binds with only 13-fold weaker affinity than A_6_, A_11_, or A_25_ and causes many of same NMR peak shifts as A_6_ (Figure 3). These data are strong evidence that the LaM of LARP1 is a stand-alone poly(A) 3′ binding domain.

Complementing the affinity measurements, we obtained high-resolution crystal structures of the LARP1 LaM in complex with seven different oligonucleotides (Figure 4). In agreement with ITC and NMR results, the crystal structures showed that RNA binding is driven by recognition of the RNA 3′-end. The position and conformation of the two 3′-adenylates were identical in all the structures while the 5′-nucleotides displayed considerable structural plasticity including two different stacking configurations (Supplementary Figure S7). His368, which is unique to LARP1, contributes to this flexibility by providing a stacking interaction with the nucleotide at position –4. The resulting –4/–1 stacking conformation likely dominates in solution although the affinity difference appears to be rather small. Loss of His368 only engenders a 4-fold loss of affinity and A_4_ binds with only 3-fold better affinity than A_3_. We further used the crystal structures to design point mutants that specifically block RNA binding. Loss of invariant Gln333, whose side chain hydrogen bonds with the adenine at position –2, decreased RNA binding affinity 20-fold with no effect on the global fold (Figure 5, Supplementary Figure S8).

The ability of LARP1 LaM to bind different RNA conformations matches the plasticity previously observed for 3′-end poly(U) sequences interacting with LARP3 (22). In LARP3, the La-module binds the UUU 3′ termini of nascent RNA polymerase III transcripts, most of which are precursors to the tRNAs, protecting them from untimely digestion by 3′ exonucleases while also assisting their folding (21,30,52). The La-module of LARP7 shares high similarity to LARP3: both recognize 3′UUU_OH_ sequences. LARP7 assembles with the 3′UUU_OH_ of 7SK non-coding nuclear RNA, to regulate activities of the positive transcription elongation factor *B* (1,5,29). The LARP6 La-module interacts with a stem-loop element in the 5′UTR of collagen mRNA to accommodate translation initiation for collagen synthesis (5,6,23). LARP4, which is most closely related to LARP1 overall but most divergent in the sequence of its LaM (1,3), binds poly(A) *in vitro* and exhibits PAT protection *in vivo* and mRNA stabilization (19,40,41,53).

The RNA binding behavior of the LARP1 LaM in the context of the full-length protein is less well-understood. While there is agreement that poly(C) RNA does not bind, a study of LARP1 (310–540) using EMSA binding assays showed a TOP20 pyrimidine-rich sequence bound with 40 nM affinity (9). Pull-down assays indicated that the LARP1 fragment is able to bind two RNA molecules simultaneously, possibly through protein oligomerization (9). Our ITC experiments with the shorter LARP1 fragment (residues 323–509) measured one-to-one binding of the same RNA with a thousand-fold weaker affinity (Figure 2B). These discrepancies suggest the regions outside of the LaM domain contribute to RNA binding despite not adopting a well-folded, static structure. While optimizing the LARP1 La-module for structural studies, we observed a propensity of larger fragments to aggregate, which could allow binding of multiple RNAs and enhance the binding affinity through avidity effects. Studies of the N-terminal region of LARP4 reported a similar behavior where regions appearing unfolded in NMR spectra and without known RNA-binding motifs bound poly(A) RNA with low micromolar affinity (19). It is tempting to speculate that RNA binding by these regions of LARP1 and LARP4 is related to their association with P-bodies and stress granules (43,54–56). The intrinsically disordered regions could drive protein and RNA binding similar to the condensation of P-bodies and stress granules through liquid-liquid phase separation (57,58).

LARP1 contains a second RNA-binding DM15 domain at its C-terminus. This domain, which is unique to LARP1 and LARP1B, binds the mRNA 5′-cap and TOP sequences to negatively regulate TOP mRNA translation in response to mTORC1 inhibition (7,10–12,15). The presence of the two RNA binding domains suggests that LARP1 and LARP1B could circularize TOP mRNAs. The LaM domain would bind the 3′-end of poly(A) while the DM15 domain would bind the 5′-end. The presence of the PABPC1-binding PAM2 motif adjacent to the LaM domain likely provides additional specificity and affinity for poly(A) RNA via recruitment of PABPC1. In some ways, the presence of PAM2 eliminates the need for LARP1 to possess a RRM domain of its own since PABPC1 contains four RNA-binding RRM domains. It is plausible that ancestral versions of LARP1 contained a RRM domain, which was subsequently replaced by the PAM2 motif.

### LARP1 and the LaM Q333A mutant provide new insight into deadenylation-mRNA decay

We used two mRNAs that are differentially engaged as substrates of the two pathway types characteristic of the biphasic kinetics model of deadenylation: β-glo-ARE a simplified unstable mRNA reporter (*t*_1/2_, ≤1.5 h), and GFP, a long-lived, stable mRNA (*t*_1/2_, ≥6 h) (59–61). The sequence-specific ARE in the β-glo-ARE mRNA mediates direct recruitment of the CNOT complex and associated deadenylases. This leads to the relatively fast decay profile of β-glo-ARE mRNA. In contrast, GFP mRNA, which lacks an ARE, is subject to the relatively slow default deadenylation pathway (reviewed in Mattijssen et al (2)).

Cumulative data in this report lead us to propose that LARP1 protection of β-glo-ARE and GFP mRNA reflects the ability of LARP1 to bind poly(A) 3′ ends and protect them from deadenylases. The PAT-seq data for these mRNAs fits with the biphasic kinetics model of deadenylation and is consistent with this interpretation. Specifically, the difference between LARP1-WT and LARP1-Q333A in PAT length protection of the GFP mRNA substrate (Figure 6E) is greater than the difference in protection observed for β-glo-ARE mRNA (Figure 6D). This suggests that LARP1 competes more effectively against default pathway deadenylation than against ARE CNOT-directed deadenylation. Conversely, the short half-life of the β-glo-ARE mRNA makes it more sensitive to LARP1 protection. We observed a large increase in β-glo-ARE mRNA levels in LARP1 expressing cells and a greater sensitivity to the Q333A mutation (Figure 6B).

In conclusion, our studies add new information and insight into the structural basis of mRNA stabilization by LARP1. Although full-length LARP1 has a mass of > 100 kDa, a single mutation in the LaM domain significantly impacts its ability to stabilize mRNA. Our previous observation that a 27 kDa fragment (residues 310-540), containing the LaM domain and PAM2 motif, was sufficient for PAT protection and mRNA stabilization highlights the central role of the LaM domain (18). The identification of point mutations that specifically block RNA binding will be useful in future studies of LARP1 activities and functions beyond mRNA stabilization.

## DATA AVAILABILITY

The coordinates were deposited into the RCSB Protein Data Bank with the accession codes: 7SOQ, 7SOR, 7SOR, 7SOS, 7SOU, 7SOT, 7SOP, 7SOV. NMR signal assignments for LARP1 LaM domain have been deposited as BMRB entry 51255.

## Supplementary Material

gkac696_Supplemental_Files
