# Supplementary material for: Structural basis of 3′-end poly(A) RNA recognition by LARP1
Source: Nucleic Acids Res. 2022 Aug 18;50(16):9534–47. doi: 10.1093/nar/gkac696 (PMC9458460; doi:10.1093/nar/gkac696)
Supplement: gkac696_Supplemental_Files [file gkac696_supplemental_files.zip › Movie captions.docx]

Supplemental Movie S1. Views of the LaM domain of LARP1 without and with different RNA ligands.

Supplemental Movie S2. Views of the LaM domain of LARP1 showing the base stacking arrangement of bound RNAs.
