# Supplementary material for: Structural basis of 3′-end poly(A) RNA recognition by LARP1
Source: Nucleic Acids Res. 2022 Aug 18;50(16):9534–47. doi: 10.1093/nar/gkac696 (PMC9458460; doi:10.1093/nar/gkac696)
Supplement: gkac696_Supplemental_Files [file gkac696_supplemental_files.zip › Suppl_material.pdf]

Supplemental material for

Structural basis of 3'-end poly(A) RNA recognition by LARP1

Guennadi Kozlov[1,2], Sandy Mattijssen[3], Jianning Jiang[1,2], Samuel Nyandwi[1,2], Tara Sprules[2,4], James R. Iben[3], Steven L. Coon[3], Sergei Gaidamakov[3], Anne M. Noronha[5], Christopher J. Wilds[5], Richard J. Maraia[3,*], Kalle Gehring[1,2,*]

[1]Department of Biochemistry & Centre for Structural Biology, McGill University, Montréal, Canada; [2]Centre de recherche en biologie structurale, McGill University, Montréal, Canada; [3]Intramural Research Program, *Eunice Kennedy Shriver* National Institute of Child Health and Human Development, National Institutes of Health, Bethesda, MD, United States; [4]Quebec/Eastern Canada NMR Centre, McGill University, Montréal, Canada; [5]Department of Chemistry and Biochemistry, Concordia University, Montréal, Canada

*E-mail: maraiar@dir6.nichd.nih.gov; kalle.gehring@mcgill.ca

Table S1. Isothermal titration calorimetry measurements

RNA	Cell, µM	Syringe, µM	Chi-Square/DofF	N	Ka (M-1)	Error (M-1)	ΔH (cal/mol)	ΔS (cal/mol/deg)	Kd (nM)	error(nM)
	**LARP1 (323-410)**									
**A_2**	30 (protein)	300 (RNA)	69100	0.808±0.0085	316000	18000.000	-23960±346	-56.5	3165	171
**A_3**	15 (RNA)	150 (protein)	534200	0.627±0.016	976000	150000	-24710±892	-56.8	1024	136
**A_3dA**	15 (RNA)	150 (protein)	488600	0.769±0.017	992000	150000	-21950±673	-47.4	1008	120
**A_4**	15 (RNA)	150 (protein)	541000	0.696±0.012	2900000	510000	-23880±669	-51.8	345	50
**A_6**	15 (RNA)	150 (protein)	137300	1.27±0.0073	4210000	410000	-17040±158	-27.8	240	24
**A_6(p)**	30 (RNA)	300 (protein)	NB	NB	NB	NB	NB	NB	NB	NB
**A_5mA**	30 (RNA)	300 (protein)	NB	NB	NB	NB	NB	NB	NB	NB
**U_6**	15 (RNA)	150 (protein)	533900	0.946±0.027	367000	51000	-27140±1110	-67.1	2725	330
**G_6**	15 (RNA)	150 (protein)	52670	0.470±0.021	305000	25000	-30120±1680	-77.6	3279	249
**C_6**	15 (RNA)	300 (protein)	NB	NB	NB	NB	NB	NB	NB	NB
**A_5U**	20 (protein)	300 (RNA)	10080	1.04±0.0079	1040000	72000	-22840±238	-50.3	962	42
**A_4UA**	20 (protein)	300 (RNA)	89200	0.932±0.0080	688000	41000	-25630±302	-60.7	1453	82
**A_3UA_2**	20 (protein)	300 (RNA)	353500	0.886±0.0084	2820000	370000	-26800±371	-61.9	297	21
**A_2UA_3**	30 (RNA)	300 (protein)	233100	1.06±00.00821	4070000	652000	-17520±208.9	-29.5	246	34
**A_5C**	20 (protein)	300 (RNA)	201500	0.411±0.057	146000	24000	-33040±5420	-89.0	6849	967
**A_4CA**	20 (protein)	300 (RNA)	121100	0.886±0.024	201000	17000	-26140±972	-64.9	4975	388
**A_3CA_2**	20 (protein)	300 (RNA)	112500	1.04±00.0050	3370000	260000	-24630±177	-54.1	297	21
**A_2CA_3**	30 (RNA)	300 (protein)	2168000	0.897±00175	5360000	1870000	-25090±749.4	-54.8	189	51
**ACA_4**	30 (RNA)	300 (protein)	1313000	1.33±00224	2740000	876000	-18260±467.3	-32.8	365	88
**A_{11}**	30 (RNA)	300 (protein)	177100	1.69±00.0131	2610000	419000	-15440±184.8	-23.3	383	53
**A_{25}**	30 (RNA)	300 (protein)	97520	1.06±0.0115	2820000	532000	-11550±186.7	-9.9	355	57
**TOP**	30 (RNA)	300 (protein)	127700	1.89±00.0863	28700	4040	-33770±2710	-94.8	～35000	4000
	**LARP1 (323-439)**									
**A_{25}**	30 (RNA)	300 (protein)	136600	0.988±0.00962	3670000	679000	-13290±196.8	-15.3	272	42
**TOP**	30 (RNA)	300 (protein)	82880	1.46±0.0524	38200	3450	-30340±1595	-82.5	26178	2000
	**LARP1 (323-509)**									
**A_{25}**	30 (RNA)	300 (protein)	40830	1.33±0.00942	4890000	868000	-7344±84.74	5.56	204	40
**TOP**	30 (RNA)	300 (protein)	11780	1.79±0.0394	29800	1600	-23160±777	-58.8	35570	3750
	**LARP1 (323-410) Q333A**									
**A_4**	30 (RNA)	300 (protein)	32580	0.482±0.017	132000	9100	-26930±1200	-68.4	7575	500
	**LARP1 (323-410) Y336A**									
**A_4**	30 (RNA)	300 (protein)	48650	0.308±0.24	43800	17000	-17580±15100	-38.7	22831	6335
	**LARP1 (323-410) R345A**									
**A_4**	30 (RNA)	300 (protein)	1349000	0.740±0.026	1260000	330000	-21250±1030	-44.5	793	5490
	**LARP1 (323-410) F348A**									
**A_4**	30 (RNA)	300 (protein)	23390	0.290±0.057	69800	9500	-24750±5530	-62.2	14327	1717
	**LARP1 (323-410) H368A**									
**A_4**	30 (RNA)	300 (protein)	125200	0.717±0.011	774000	69000	-19710±428	-40.3	1290	108
	**LARP1 (323-410) Q333A, F348A**									
**A_4**	30 (RNA)	300 (protein)	NB	NB	NB	NB	NB	NB	NB	NB
	**LARP1 (323-410) Y336A, F348A**									
**A_4**	30 (RNA)	300 (protein)	NB	NB	NB	NB	NB	NB	NB	NB
	**LARP1 (323-509) Q333A, F348A**									
**A_{25}**	15 (RNA)	150 (protein)	NB	NB	NB	NB	NB	NB	NB	NB
	**LARP1 (323-509) Y336A, F348A**									
**A_{25}**	15 (RNA)	150 (protein)	NB	NB	NB	NB	NB	NB	NB	NB

Error estimates are from thermogram fitting
NB: no binding

Table S2. Data Collection and Refinement Statistics

**Data collection**	LaM	LaM-AAA	LaM-AAA	LaM-AAAA
PDB code	7SOO	7SOQ	7SOR	7SOS
Space group	$P2_12_12_1$	$P2_12_12_1$	$P2_12_12_1$	$P2_12_12_1$
Cell dimensions				
a, *b*, *c* (Å)	36.57, 46.78, 57.01	36.92, 46.47, 59.13	48.56, 55.89, 70.17	36.19, 46.80, 58.74
Resolution (Å)	50-1.65 (1.71-1.65)	50-1.15 (1.17-1.15)	50-1.35 (1.37-1.35)	50-1.25 (1.27-1.25)
R_{sym}	0.049 (0.340)	0.061 (0.431)	0.039 (0.633)	0.039 (0.464)
$I / \sigma I$	55.9 (3.7)	32.2 (2.7)	44.5 (1.6)	50.4 (1.2)
Completeness (%)	98.7 (93.8)	96.8 (75.6)	99.8 (97.9)	97.7 (71.6)
Redundancy	7.4 (5.7)	8.0 (4.8)	8.3 (6.8)	7.0 (2.9)
CC1/2[2]	0.888	0.840	0.918	0.809
**Refinement**				
Resolution (Å)	36.2 - 1.65	36.5 - 1.15	43.7 - 1.35	36.6 - 1.25
No. reflections	12117	35581	42380	28289
R_{work} / R_{free}	0.205/0.224	0.175/0.188	0.180/0.204	0.174/0.195
No. atoms				
Protein	724	765	1472	727
RNA		63	124	76
Water	48	115	157	100
Nucleotide				
B-factors				
Protein	26.0	15.2	19.5	20.5
RNA		19.6	29.8	23.8
Water	28.3	27.7	27.9	32.9
Nucleotide				
R.m.s deviations				
Bond lengths (Å)	0.005	0.014	0.014	0.016
Bond angles (°)	0.79	1.43	1.58	1.67
Ramachandran statistics (%)				
Most favored regions	97.7	97.7	98.3	97.7
Additional allowed regions	2.3	2.3	1.7	2.3
Disallowed regions	0.0	0.0	0.0	0.0

[1]Highest resolution shell is shown in parentheses.
[2]CC1/2 in highest resolution shell.

Table S2 (continued)

**Data collection**	LaM-AAAUAA	LaM-AAAAAA	LaM-AAAAAA	LaM-AAAAAAAAAAA
PDB code	7SOP	7SOT	7SOU	7SOV
Space group	$P2_12_12_1$	$P2_12_12_1$	$P2_12_12_1$	$P2_12_12_1$
Cell dimensions				
a, *b*, *c* (Å)	36.52, 46.26, 57.37	36.67, 46.38, 58.62	36.87, 46.26, 58.92	36.78, 46.31, 58.63
Resolution (Å)	50-1.55 (1.58-1.55)	50-1.53 (1.56-1.53)	50-1.45 (1.48-1.45)	50-1.45 (1.48-1.45)
R_{sym}	0.057 (0.313)	0.039 (0.565)	0.086 (0.507)	0.086 (0.595)
$I / \sigma I$	29.0 (5.4)	46.7 (2.6)	19.0 (2.2)	18.8 (2.5)
Completeness (%)	98.6 (83.0)	99.4 (100)	98.9 (96.9)	95.8 (98.7)
Redundancy	6.6 (2.8)	7.3 (7.2)	7.3 (6.2)	6.6 (7.1)
CC1/2[2]	0.753	0.776	0.915	0.846
**Refinement**				
Resolution (Å)	36.0 - 1.55	36.4 - 1.52	36.4 - 1.45	36.3 - 1.45
No. reflections	14361	15675	18268	17326
R_{work} / R_{free}	0.194/0.220	0.190/0.211	0.183/0.202	0.183/0.223
No. atoms				
Protein	723	749	738	732
RNA	69	66	62	79
Water	60	69	93	63
Nucleotide				
B-factors				
Protein	31.2	25.1	19.8	25.5
RNA	41.9	39.4	29.8	45.2
Water	38.1	34.4	30.4	35.5
Nucleotide				
R.m.s deviations				
Bond lengths (Å)	0.013	0.012	0.017	0.009
Bond angles (°)	1.35	1.29	1.89	1.26
Ramachandran statistics (%)				
Most favored regions	97.7	97.7	97.7	97.7
Additional allowed regions	2.3	2.3	2.3	2.3
Disallowed regions	0.0	0.0	0.0	0.0

[1]Highest resolution shell is shown in parentheses.

[2]CC1/2 in highest resolution shell.

Table S3. Structural parameters of bound RNAs

**Glycosidic angle (χ)**				Residue	
Ligand	PDB	stacking	-4/-3	-2	-1
A3	7SOQ	-3/-1	57	-110	-119
A3 chain B	7SOR	-3/-1	-86	-111	-133
A3 chain D	7SOR	-3/-1	-90	-109	-130
A4	7SOS	-4/-1	53	-109	-130
A6	7SOU	-3/-1	35	-112	-122
A6	7SOT	-4/-1	49	-112	-125
A3UA2	7SOP	-4/-1	57	-111	-115
A11	7SOV	-4/-1	47	-113	-124

**Pseudorotation angle**				Residue	
Ligand	PDB	stacking	-4/-3	-2	-1
A3	7SOQ	-3/-1	160	157	157
A3 chain B	7SOR	-3/-1	16	162	142
A3 chain D	7SOR	-3/-1	14	162	135
A4	7SOS	-4/-1	162	164	119
A6	7SOU	-3/-1	41	159	150
A6	7SOT	-4/-1	164	166	128
A3UA2	7SOP	-4/-1	169	162	147
A11	7SOV	-4/-1	165	165	135

**Sugar pucker**				Residue	
Ligand	PDB	stacking	-4/-3	-2	-1
A3	7SOQ	-3/-1	39	41	34
A3 chain B	7SOR	-3/-1	28	40	41
A3 chain D	7SOR	-3/-1	28	39	42
A4	7SOS	-4/-1	36	41	35
A6	7SOU	-3/-1	21	40	30
A6	7SOT	-4/-1	37	41	30
A3UA2	7SOP	-4/-1	39	39	33
A11	7SOV	-4/-1	37	41	33

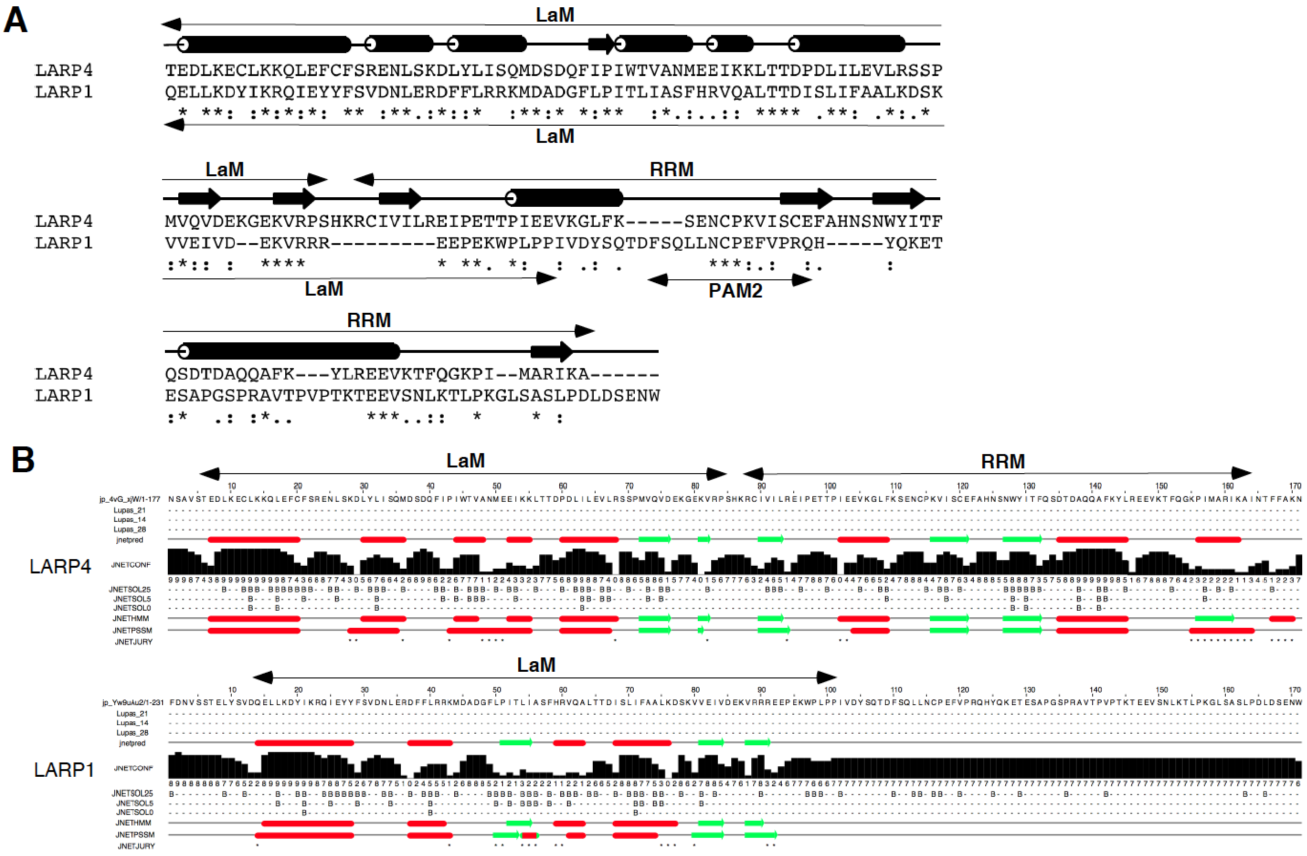

Figure S1. Comparison of La Module regions in LARP1 and LARP4. (A) Sequence alignment of LARP4 La Module with the corresponding region of LARP1 shows high conservation of the LaM domain and much lower sequence similarity in the downstream RRM region. The LARP1 LaM domain is immediately followed by a PAM2 motif. Secondary structure elements from LARP4 La-module structure (PDB 6I9B) are shown above the alignment. **(B)** JPred4 secondary structure prediction for both LaM and RRM regions of LARP4 is in a good agreement with experimentally determined structure. The RRM region of LARP1 is devoid of predicted secondary structure elements.

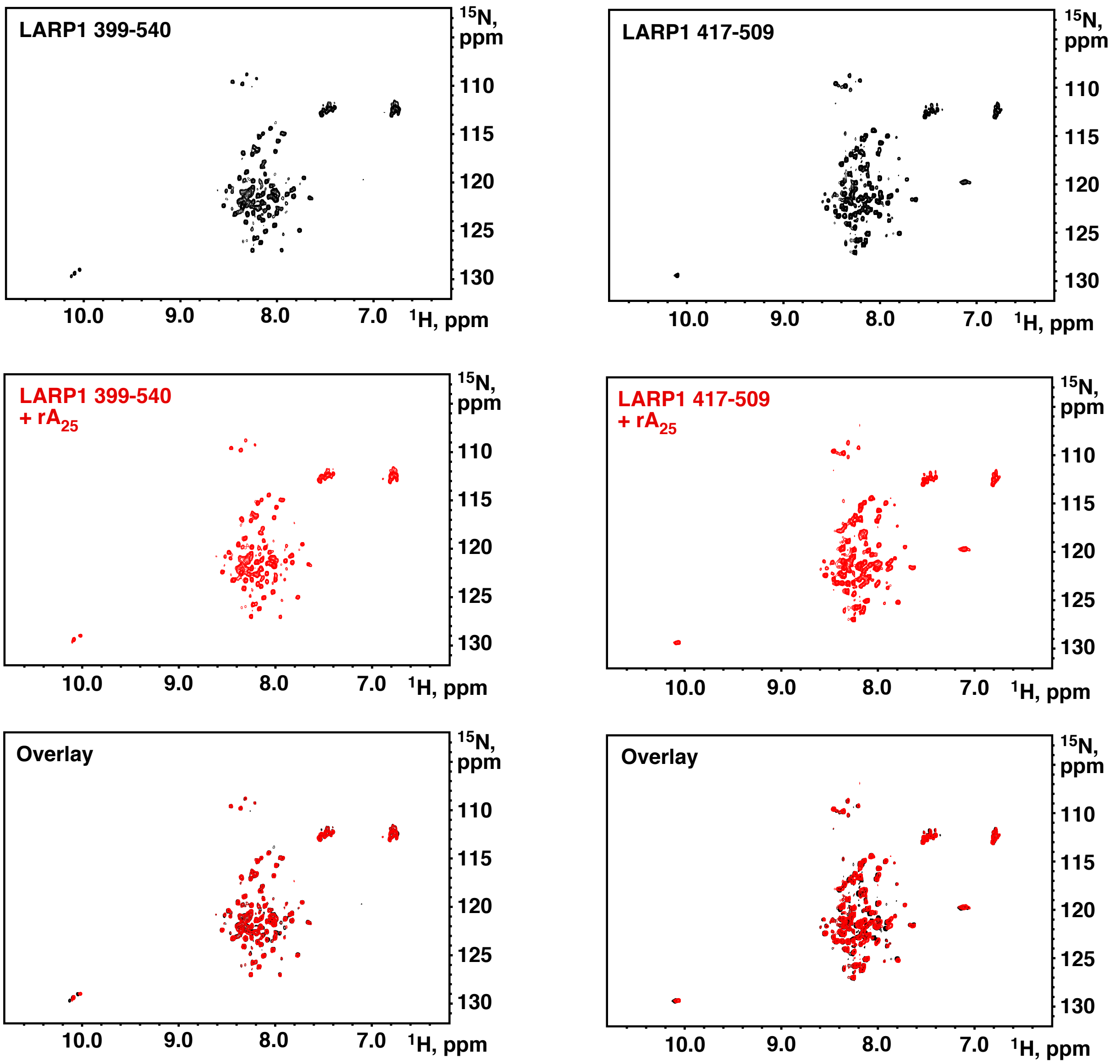

Figure S2. C-terminal extension of the putative RRM or addition of RNA do not promote folding of the LARP1 RRM region. ^{1}H-^{15}N NMR correlation spectra for LARP1 fragments comprising residues 399-540 (left column) and residues 417-509 (right column). Spectra of ^{15}N-labeled LARP1fragments alone (50 µM) are shown in black and in the presence of A_{25} RNA (50 µM) are in red. The absence of chemical shift changes upon addition of RNA indicates no binding.

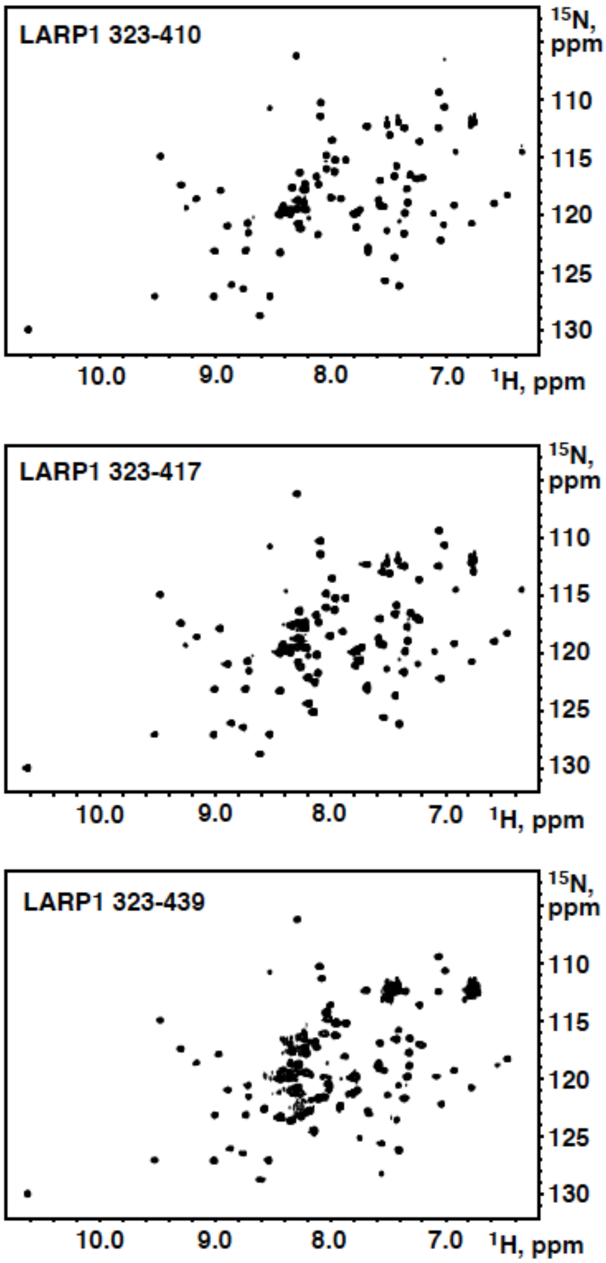

Figure S3. Identification of the LARP1 LaM domain C-terminal boundary. ^{15}N-^{1}H correlation spectrum of ^{15}N-labeled LARP1 (323-410) shows presence of a well-folded domain characterized by good dispersion of signals. The spectra of longer LARP1 constructs 323-417 and 323-439 show an increased number of strong signals in the middle of the spectrum indicating the presence of unstructured residues.

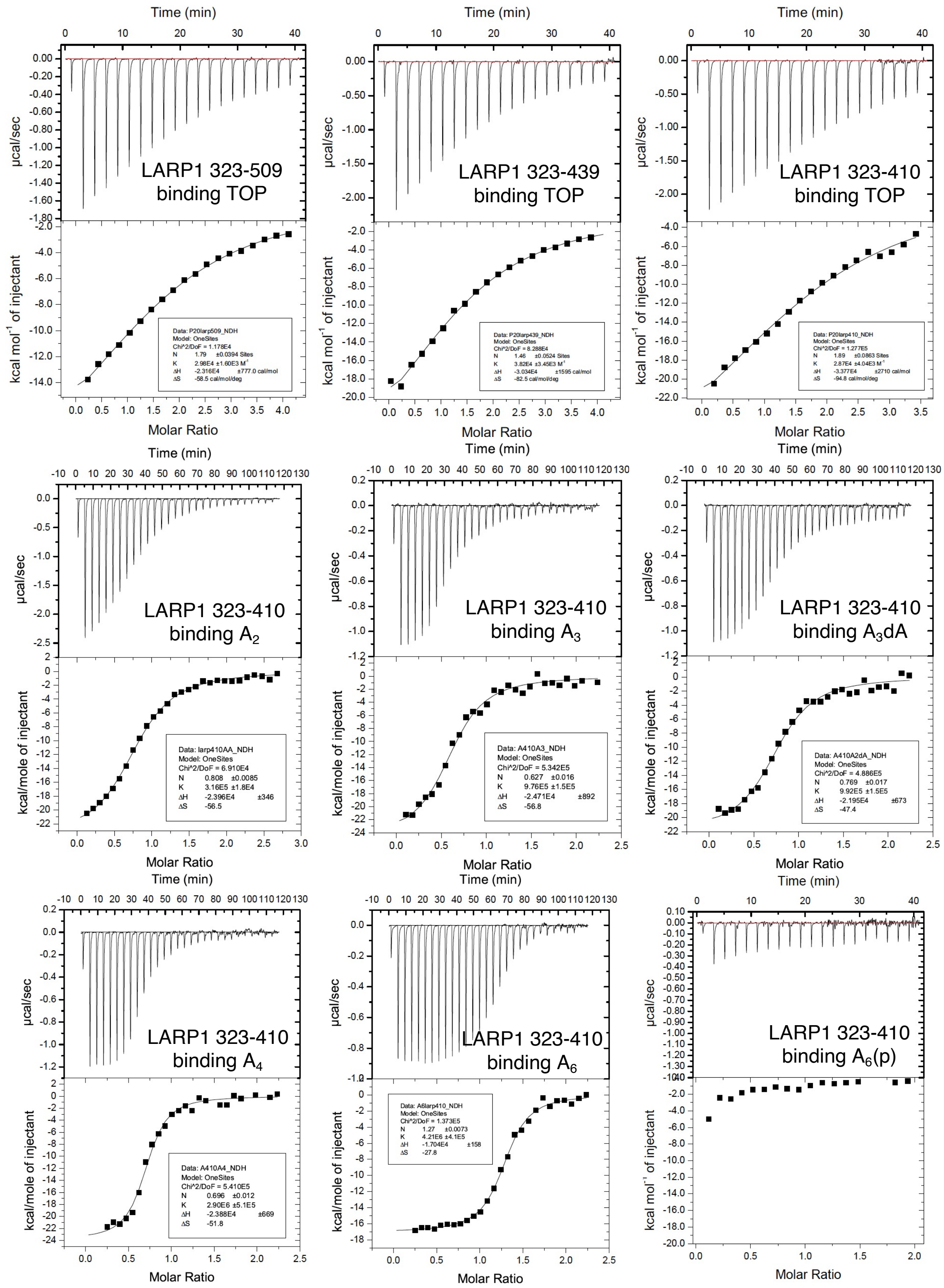

Figure S4 (continued)

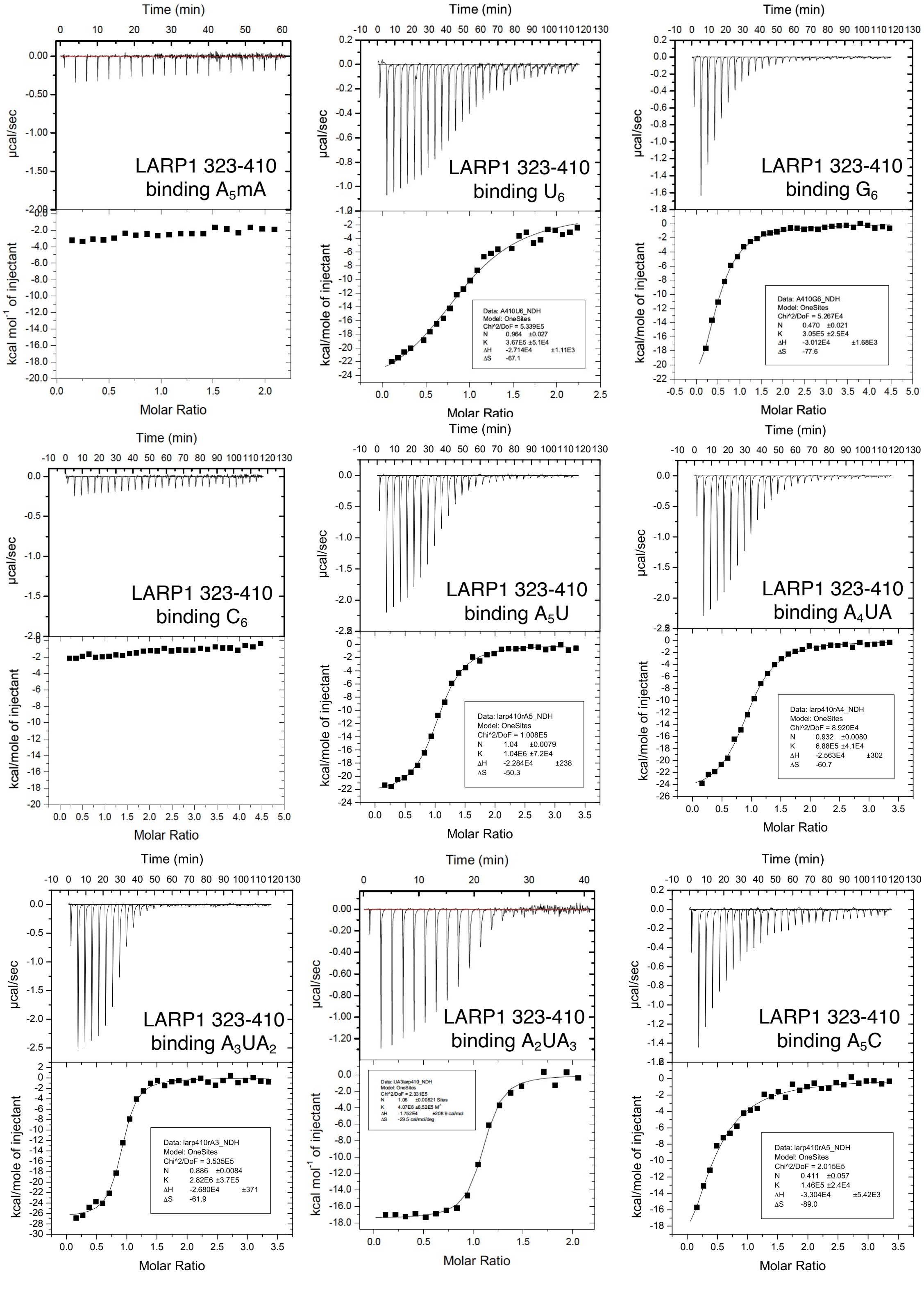

Figure S4 (continued)

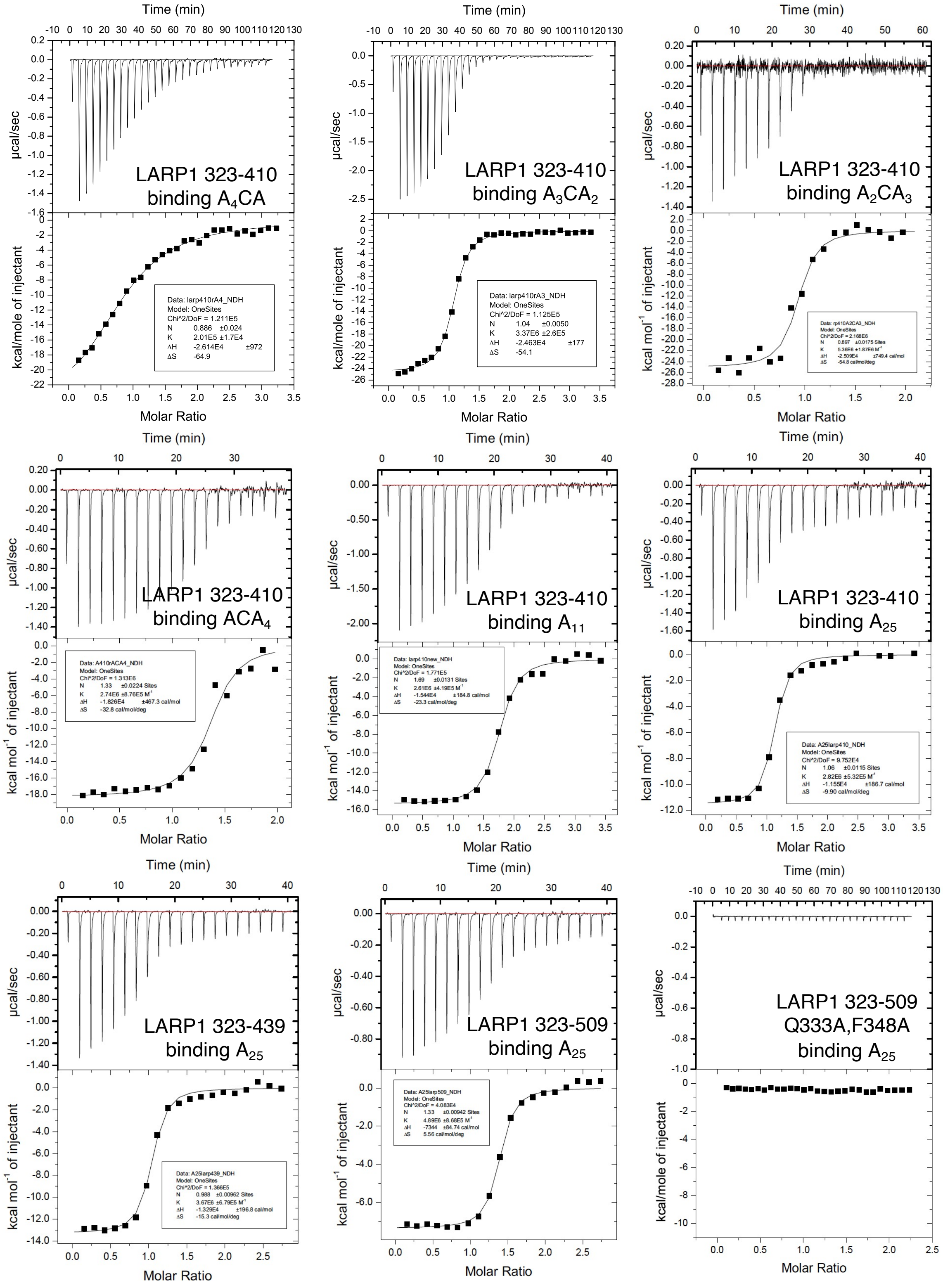

Figure S4 (continued)

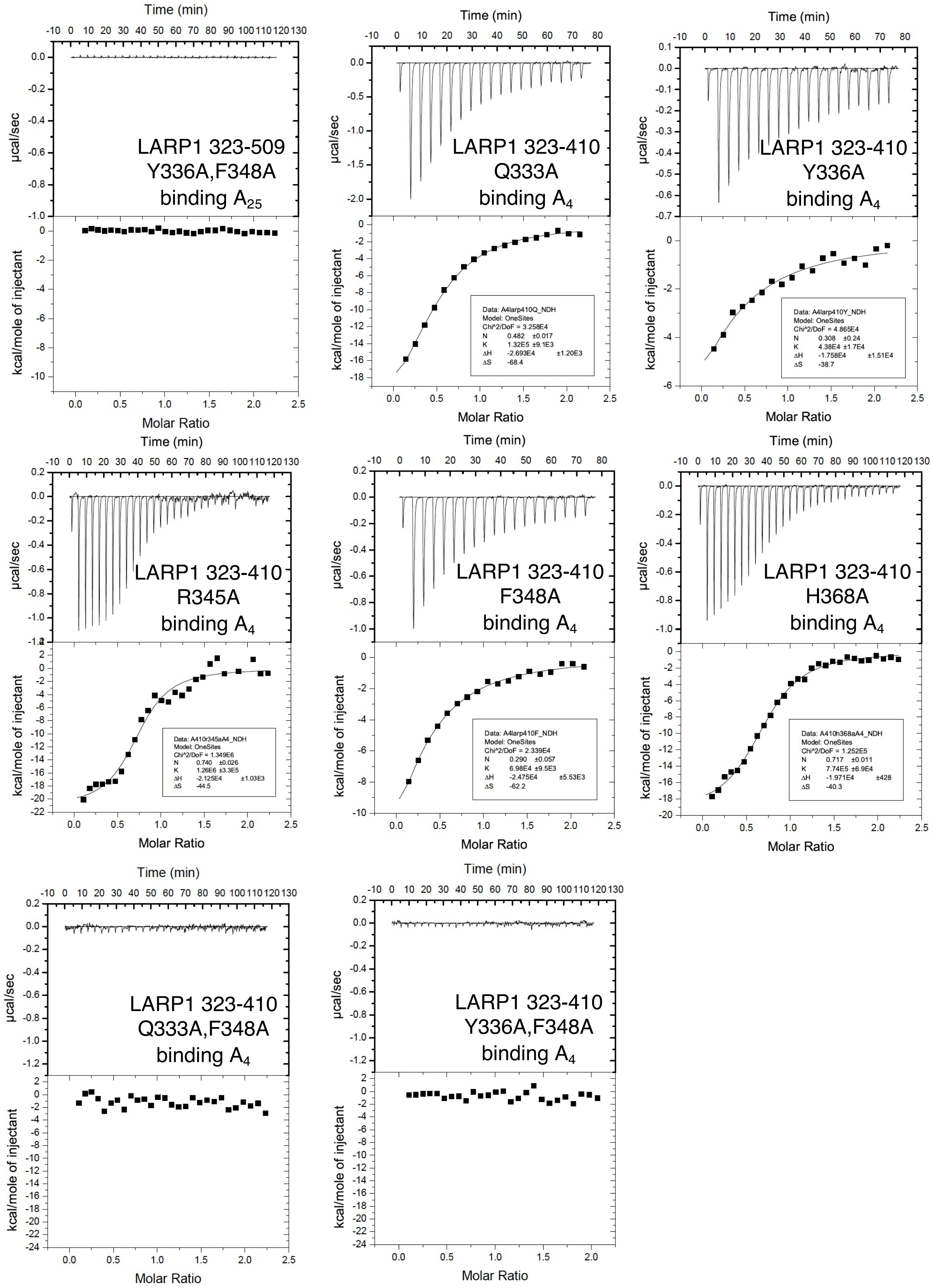

Figure S4. ITC thermograms of binding between LARP1 fragments and RNAs.

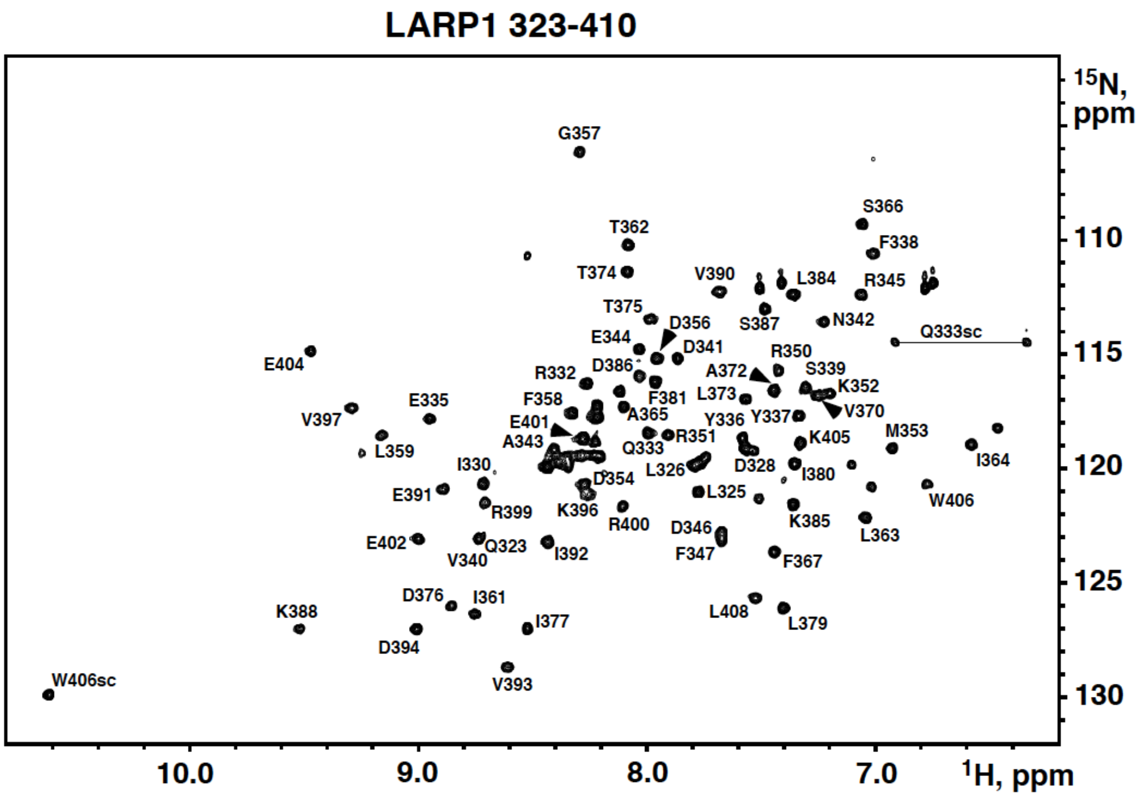

Figure S5. Peak assignments of the ^{15}N-^{1}H correlation spectrum of ^{15}N-labeled LARP1 (323-410). Assignments were done by standard heteronuclear 3D NMR experiments using ^{13}C,^{15}N-labeled protein and deposited at the Biological Magnetic Resonance Data Bank (BMRB) as entry 51255. The side chains resonances of W406 and Q333 are labeled *sc*.

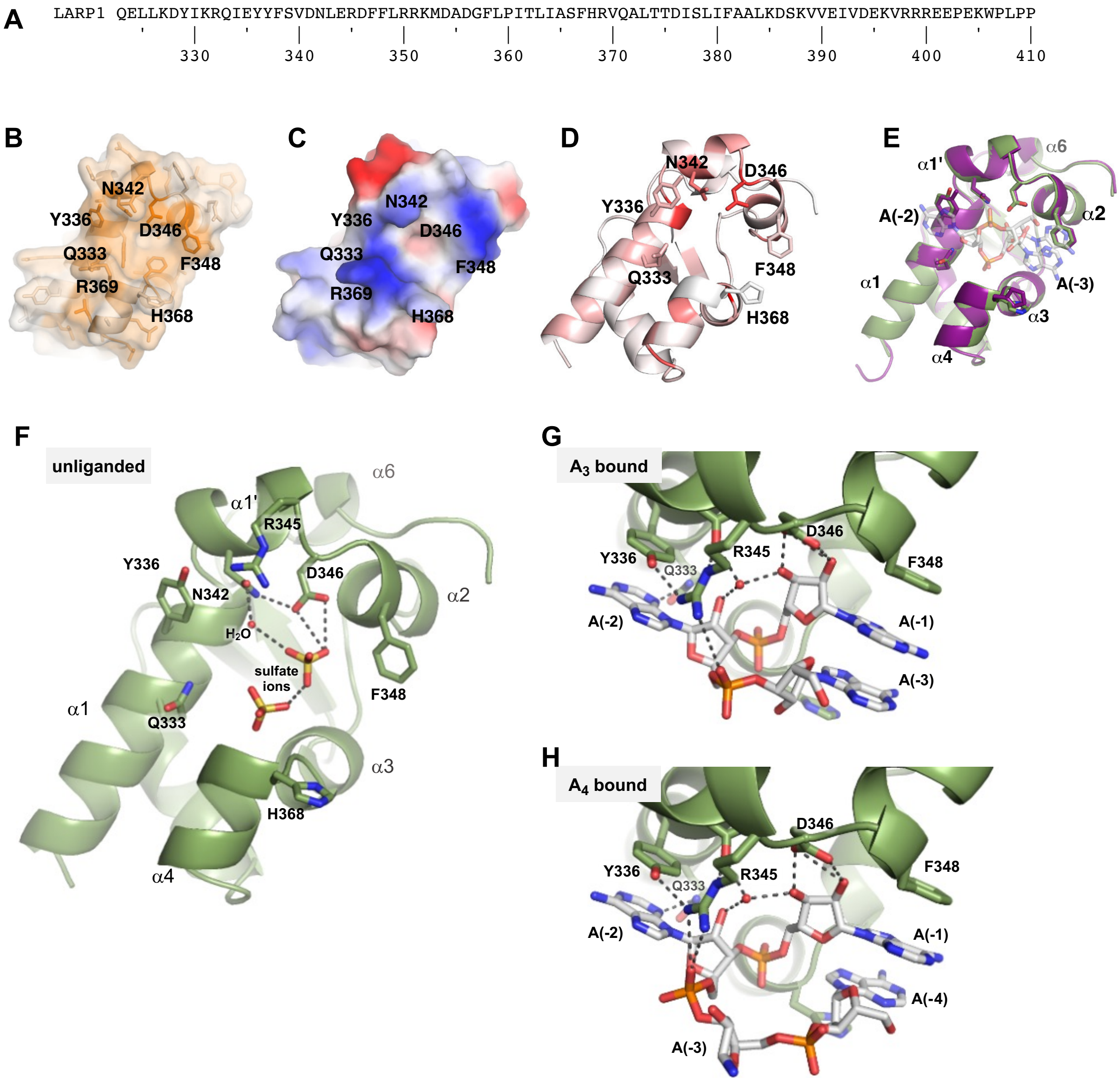

Figure S6. Structure of the LARP1 LaM domain. (A) Amino acid sequence of LARP1 LaM, numbered according to the 1019 amino acid long isoform. **(B)** Conservation of LaM domain residues across human LARP proteins, color-coded from high (*orange*) to low conservation (*pale*). **(C)** Electrostatic surface of LARP1 LaM domain. **(D)** NMR chemical shift changes in LaM upon addition of 50 µM A_6, color-coded from large (*red*) to no shift (*white*). **(E)** Overlay of unliganded (*magenta*) and A_3-bound (*green*) LARP1 LaM structures shows only minor structural changes upon RNA binding. **(F)** Structure of unliganded LaM including Arg345 which helps position an RNA phosphate in the bound structures. **(G)** Structure of LaM with A_3 RNA bound. **(H)** Structure of LaM with A_4 bound.

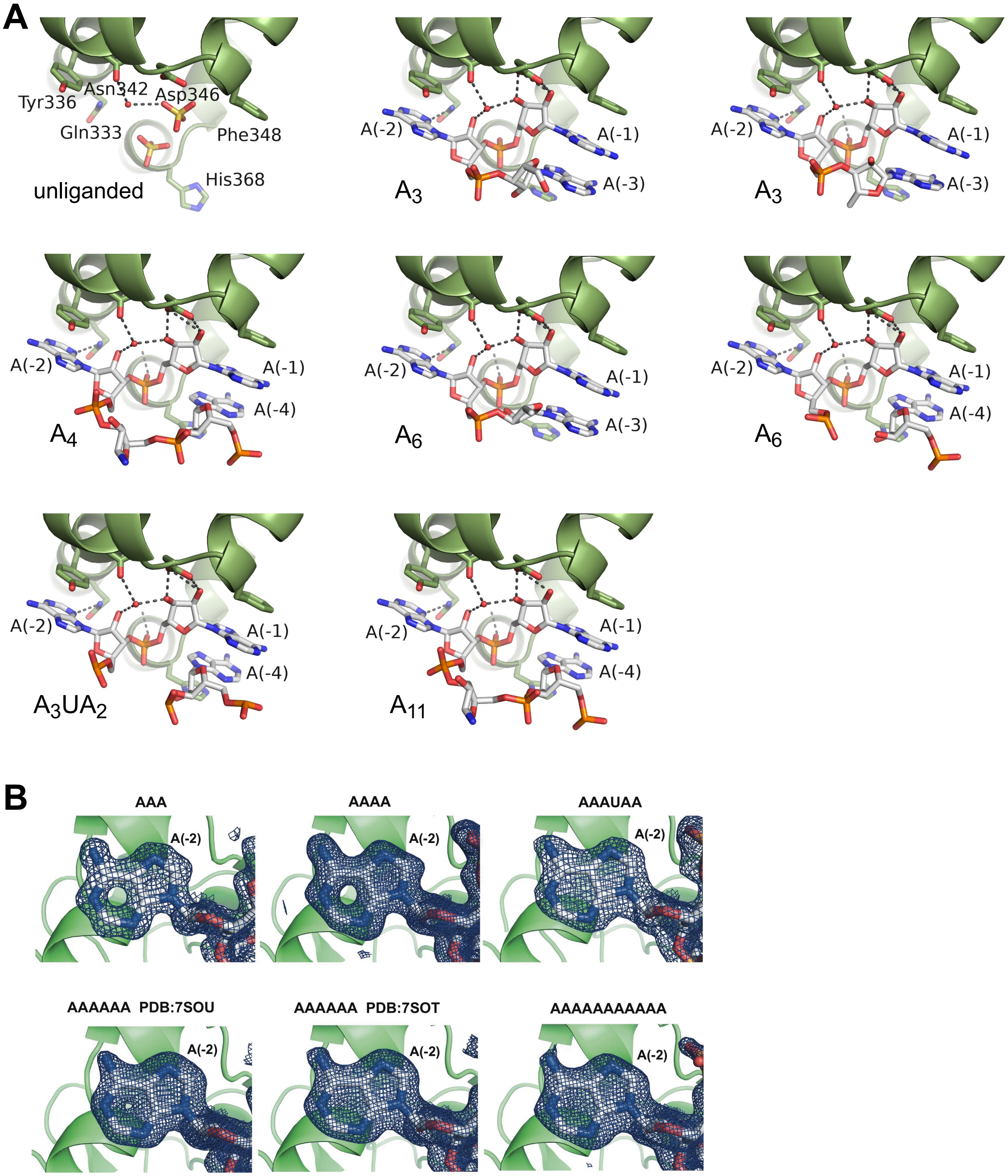

Figure S7. LARP1 LaM crystal structures. (A) Structures without and with RNA bound. **(B)** Representative electron density of the penultimate A(-2) adenine base contoured at 1σ from RNA $2F_O$-F_C omit maps.

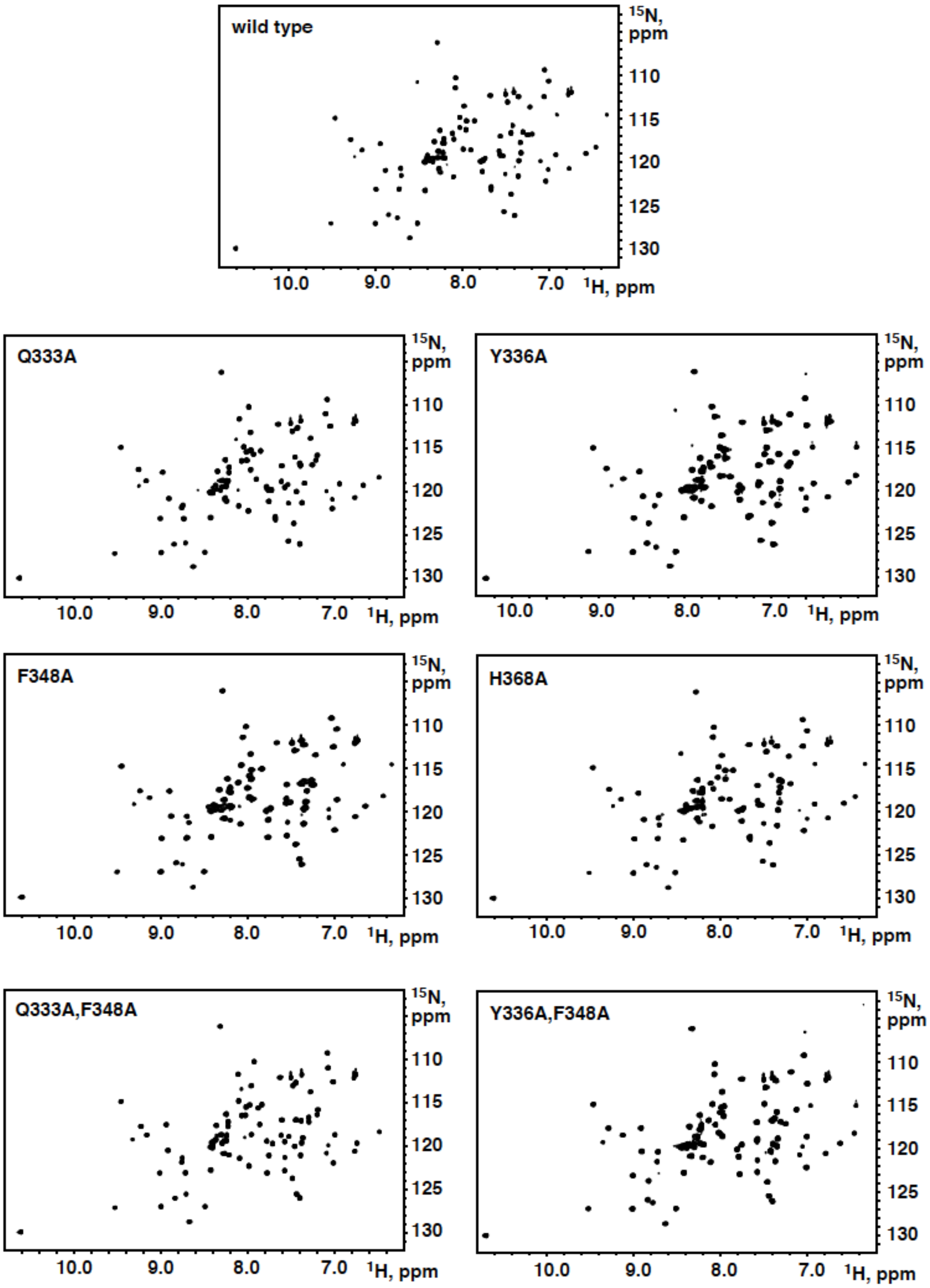

Figure S8. Verification of the structural integrity of the LaM domain mutants by NMR. ^{15}N-^{1}H correlation spectra of ^{15}N-labeled LARP1 (323-410) single and double mutants are very similar to the wild type spectrum, confirming that the mutations did not unfold the domain. We were not able to produce a D346I mutant as the mutation destabilized the domain
